# The sugar‐responsive circadian clock regulator bZIP63 modulates plant growth

**DOI:** 10.1111/nph.17518

**Published:** 2021-06-30

**Authors:** Américo J. C. Viana, Cleverson C. Matiolli, David W. Newman, João G. P. Vieira, Gustavo T. Duarte, Marina C. M. Martins, Elodie Gilbault, Carlos T. Hotta, Camila Caldana, Michel Vincentz

**Affiliations:** ^1^ Centro de Biologia Molecular e Engenharia Genética Departamento de Biologia Vegetal Instituto de Biologia Universidade Estadual de Campinas CEP 13083‐875, CP 6010 Campinas SP Brazil; ^2^ Brazilian Bioethanol Science and Technology Laboratory (CTBE/CNPEM) Rua Giuseppe Máximo Scolfaro 10000 Campinas SP CEP 13083‐970 Brazil; ^3^ Max‐Planck Partner Group Brazilian Bioethanol Science and Technology Laboratory (CTBE/CNPEM) Campinas, SP Brazil; ^4^ Laboratory of Plant Physiological Ecology Department of Botany Institute of Biosciences University of São Paulo São Paulo, SP CEP 05508‐090 Brazil; ^5^ Institut Jean‐Pierre Bourgin INRAE AgroParisTech Université Paris‐Saclay Versailles 78000 France; ^6^ Departamento de Bioquímica Instituto de Química Universidade de São Paulo São Paulo, SP CEP 05508‐000 Brazil; ^7^ Max Planck Institute of Molecular Plant Physiology Am Mühlenberg 1 14476 Potsdam Golm Germany

**Keywords:** Arabidopsis, bZIP63, circadian clock, growth, low energy stress, starch

## Abstract

- Adjustment to energy starvation is crucial to ensure growth and survival. In *Arabidopsis thaliana* (Arabidopsis), this process relies in part on the phosphorylation of the circadian clock regulator bZIP63 by SUCROSE non‐fermenting RELATED KINASE1 (SnRK1), a key mediator of responses to low energy.
- We investigated the effects of mutations in *bZIP63* on plant carbon (C) metabolism and growth. Results from phenotypic, transcriptomic and metabolomic analysis of *bZIP63* mutants prompted us to investigate the starch accumulation pattern and the expression of genes involved in starch degradation and in the circadian oscillator.
- *bZIP63* mutation impairs growth under light‐dark cycles, but not under constant light. The reduced growth likely results from the accentuated C depletion towards the end of the night, which is caused by the accelerated starch degradation of *bZIP63* mutants. The diel expression pattern of *bZIP63* is dictated by both the circadian clock and energy levels, which could determine the changes in the circadian expression of clock and starch metabolic genes observed in *bZIP63* mutants.
- We conclude that *bZIP63* composes a regulatory interface between the metabolic and circadian control of starch breakdown to optimize C usage and plant growth.

## Introduction

Plants rely on a sophisticated network of metabolic and environmental receptors that trigger downstream signaling pathways to cope efficiently with environmental changes and ensure survival. Efficient management of nutrient and energy resources (i.e. mainly carbohydrates but also amino acids and fatty acids, all of which can fuel respiration to produce ATP) is crucial to accommodate growth, development and stress responses, counting on the adequate regulation of anabolic and catabolic processes (Baena‐González *et al*., 2007; Baena‐González & Sheen, 2008; Robaglia *et al*., 2012; Lastdrager *et al*., 2014; Tomé *et al*., 2014). The balance between anabolism and catabolism is regulated mostly by the antagonistic activity of two evolutionary conserved kinases, namely *SUCROSE non‐fermenting RELATED KINASE1* (*SnRK1*) and *TARGET OF RAPAMYCIN* (*TOR*), which repress and activate growth under conditions of low and high energy levels, respectively (Baena‐González *et al*., 2007; Robaglia *et al*., 2012; Dobrenel *et al*., 2016). The usage of photosynthetically produced carbohydrates in Arabidopsis is tightly controlled to maintain a steady supply of energy (Gibon *et al*., 2004; Graf *et al*., 2010; Stitt & Zeeman, 2012). Photosynthates are partitioned into sucrose and transitory starch, which is mostly consumed during the dark period to sustain metabolic activities and growth (Lu *et al*., 2005; Smith & Stitt, 2007; Graf *et al*., 2010; Kölling *et al*., 2015).

In Arabidopsis, dynamic adjustment of the starch degradation requires a functional circadian clock to ensure proper supply of carbohydrates until dawn (Gibon *et al*., 2004; Baena‐González *et al*., 2007; Graf *et al*., 2010; Scialdone *et al*., 2013; Seki *et al*., 2017), but the molecular players and the mechanisms involved in this regulation are mostly unknown. The plant circadian clock provides a way to predict daily and seasonal environmental changes associated with the movements of the Earth (i.e. rhythmic changes in light availability and temperature), allowing optimization of metabolic activities, development, growth and responses to stress (Green *et al*., 2002; Dodd *et al*., 2005; Harmer, 2009; Seo & Mas, 2015; Shim & Imaizumi, 2015). Mathematical models have been proposed to account for the empirical data of starch turnover dynamics regulated by the circadian clock. To describe the mechanisms by which the appropriate rate of starch degradation is set by the circadian clock, some models focus on the detection of the starch amount (Scialdone *et al*., 2013; Pokhilko *et al*., 2014; Scialdone & Howard, 2015), whereas others focus on the perception and maintenance of sucrose homeostasis (Haydon *et al*., 2013; Seki *et al*., 2017).

We have shown that the Arabidopsis transcription factor bZIP63, which is a key target of SnRK1, regulates some of the transcriptional changes induced by energy deprivation (Baena‐González *et al*., 2007; Matiolli *et al*., 2011; Mair *et al*., 2015) and also mediates the circadian clock entrainment by sugars through regulation of *PSEUDO‐RESPONSE REGULATOR 7* (*PRR7*) transcription (Frank *et al*., 2018). Here we present evidence that both the circadian clock and the energy status regulate *bZIP63* transcript accumulation. This dual regulation of *bZIP63* expression may, in turn, affect its target genes expression, including those involved in starch degradation and energy deficit responses. Accordingly, *bZIP63* mutants displayed alteration in starch degradation and impaired growth in diel cycles. Our results suggest that *bZIP63* is a link between energy status and the circadian clock to control starch degradation and responses to energy stress, fine‐tuning carbon utilization through the diel cycle.

## Materials and Methods

### Plant material and growth conditions

*Arabidopsis thaliana* Wassilewskija (Ws) and Columbia‐0 (Col‐0) ecotypes, as well as the T‐DNA insertion mutants *bzip63‐2* (FLAG_610A08; Matiolli *et al*., 2011; Supporting Information Fig. S1a–d) and *bzip63‐3* (FLAG_532A10; Fig. S1a,b), were obtained from the Arabidopsis Biological Resource Center (ABRC). The mutants *cca1‐11/lhy‐21* (*cca1/lhy*; ABRC germplasm CS9380, Ws background; Graf *et al*., 2010) (CCA1, CIRCADIAN CLOCK ASSOCIATED 1; LHY, Late Elongated Hypocotyl), *phosphoglucomutase* (*pgm*) (Col‐0 background; Gibon *et al*., 2004), and the overexpressing lines *HA‐bZIP63*‐ox1 and *HA‐bZIP63*‐ox2 (Frank *et al*., 2018) were described previously. In all experiments, plants were grown in a 2:1 mix of substrate (Plantmax HT, São Paulo, Brazil) and fine vermiculite (Plantmax). Sown seeds were kept in the dark at 4°C for 72 h to break dormancy, and afterwards were grown at 22°C and Photosynthetically Active Radiation (PAR) of 100 μmol m^−2^ s^−1^ under short day (SD, 10 h : 14 h, light : dark), long day (LD, 16 h : 8 h, light : dark), equinoctial (12 h : 12 h, light : dark) or free‐running (LL) photoperiods.

For transcriptome analysis and validation of misregulated genes by quantitative reverse transcription PCR (qRT‐PCR) analysis, total RNA was extracted from the four youngest leaves and the shoot apical meristems of plants grown under SD conditions for 25 d, and harvested at the end of the night (EN) immediately before the onset of the light, because *bZIP63* is most expressed in meristem and young leaves (Weltmeier *et al*., 2009) and at EN (DIURNAL Database: http://diurnal.mocklerlab.org; Fig. S1e). For chromatin immunoprecipitation (ChIP) analysis, 1 g of the entire aerial part of plants grown under equinoctial conditions for 12 d were harvested at EN. For gene expression analyses under diel and free‐running conditions, plants were grown under equinoctial conditions for 30 d. Then, samples from the entire aerial part were harvested every 4 h for 2 d, whereas another set of plants was released into free‐running conditions for 24 h before being sampled every 4 h for 48 h (24–72 h, LL) and snap‐frozen in liquid N_2_ for subsequent total RNA extraction. For metabolic profiling analysis, plants were grown under equinoctial conditions for 25 d and entire aerial parts were harvested at the end of the night immediately before the onset of the light. Each biological replicate was composed of a pool sampled from five plants for gene expression analysis or six plants for metabolic profiling analysis. Phenotypic analyses were performed in 30‐d‐old plants grown under SD, LD, equinoctial or LL conditions, and in Phenoscope platform (https://phenoscope.versailles.inra.fr; Tisné *et al*., 2013) plants grown under 8 h : 16 h, light : dark photoperiod.

### RNA isolation, cDNA synthesis and qRT‐PCR analysis

Total RNA for the transcriptome analysis was isolated using the RNeasy Plant Mini kit (Qiagen) according to the manufacturer’s instructions. The purity of total RNA samples was verified by spectrophotometry (260/230 and 260/280 ratios ≥ 1.8; NanoVue, GE Healthcare Life Sciences, Piscataway, NJ, USA). RNA integrity was evaluated by capillary electrophoresis (RNA integrity number > 8, Bioanalyzer 2100; Agilent, Santa Clara, CA, USA). Total RNA isolation for qRT‐PCR was performed as described previously (Oñate‐Sánchez & Vicente‐Carbajosa, 2008), with modifications (Oliveira *et al*., 2015). For gene expression quantification of intron‐less genes, RNA was treated with Ambion^®^ Turbo DNA‐free DNAse (cat. no. AM1907; Thermo Fisher Scientific, Waltham, MA, USA) following the manufacturer’s instructions. cDNA synthesis from 1.5 µg total RNA (final volume 12.5 ml) was performed using ImProm II Reverse Transcriptase (cat. no. A3802; Promega) and oligo(dT)_18_ according to the manufacturer’s instructions. qRT‐PCR analyses were performed as described previously (Matiolli *et al*., 2011), using Platinum SYBR green (cat. no. 11733‐038; Invitrogen), and run on a 7500 Fast Real‐Time PCR System (Applied Biosystems, Foster City, CA, USA). *PP2AA3* (AT1G13320) or *ACTIN2* (AT3G18780) were used as reference genes as indicated in the text (Czechowski *et al*., 2005).

### Microarray and data analysis

**Fig. 1:**
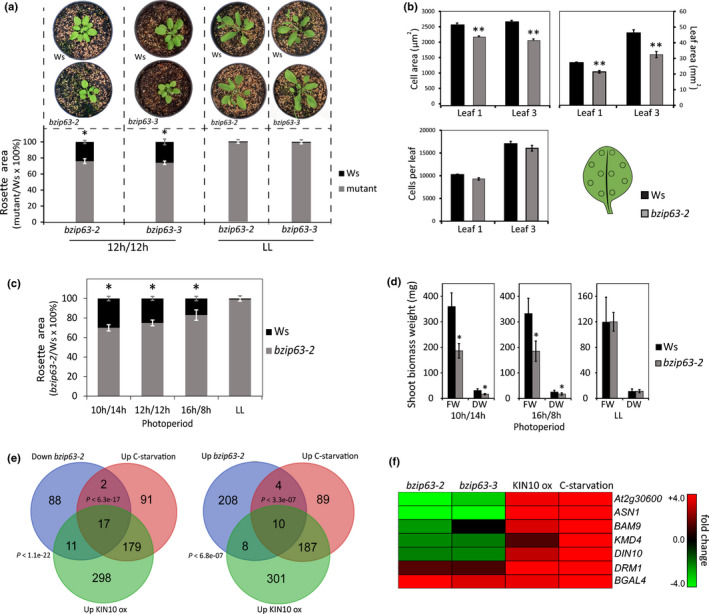
*Arabidopsis bZIP63* mutants present reduced growth under diel cycles. (a) The mutants *bzip63‐2* and *bzip63*‐3 exhibit reduced rosette area when grown under diel cycles (12 h : 12 h, light : dark photoperiod). The rosette area of *bZIP63* mutants and Wassilewskija (Ws) were indistinguishable when plants were grown under free‐running conditions (LL). (b) Leaf epidermal cell properties of *bzip63‐2* compared to Ws grown under diel cycles (12 h : 12 h, light : dark photoperiod). Leaf area was measured directly whereas the number of cells in the leaf epidermis and epidermal cell area were calculated from a series of measurements corresponding to *c.* 10% of the total leaf area taken from 10 different positions across the leaf using an Eclipse 80i light microscope, and displayed as the mean of 10 samples obtained from two independent experiments with the corresponding SE values. Leaf order was counted as the oldest leaf developed after the cotyledons onwards (two‐tailed Student’s *t*‐test; *n* = 20; **, *P* < 0.01). (c) Differences in rosette area between *bzip63‐2* and Ws are inversely correlated to photoperiod length, where short days (SD; 10 h : 14 h, light : dark photoperiod) unveil the most contrasting rosette areas between *bzip63‐2* and Ws. The data were obtained from three and two independent experiments for *bzip63‐2* and *bzip63‐3*, respectively, with 30 biological replicates for each genotype (two‐tailed Student’s *t*‐test; *n* = 90 for *bzip63‐2* and 60 for *bzip63‐3*; *, *P* < 0.05). Values are the percentage of mean rosette area in *bZIP63* mutants compared to Ws, and error bars indicate standard deviation. (d) FW and DW differences between *bzip63‐2* and Ws are inversely correlated to photoperiod length, where SDs promoted the most contrasting shoot biomass weight. Under LL no difference for both FW and DW was observed between genotypes. Plants were grown for 30 d under photoperiods or 25 d under LL. The biomass weight was obtained from two independent experiments, with 30 biological replicates each (Student’s *t*‐test; *n* = 60; *, *P* < 0.05). Values are means, and error bars indicate standard deviation. (e) Overlap between genome‐wide misregulated genes in *bzip63‐2*, genes induced by KIN10 overexpression (KIN10 ox; Baena‐González *et al*., 2007) and various carbon (C) starvation conditions (Contento *et al*., 2004; Gibon *et al*., 2004; Usadel *et al*., 2008; Cookson *et al*., 2016). (f) Clustering of energy stress associated genes misregulated in *bzip63‐2*, induced by KIN10 ox and by various C starvation conditions and selected for validation in *bzip63‐3* through quantitative reverse transcription PCR.

Three biological replicates of each genotype (Wassilewskija (Ws) and *bzip63‐*2) were used for transcriptome analysis. Each replicate was composed of a pool of the four youngest leaves and shoot apical meristem harvested from five plants. Hybridization on the GeneChip Arabidopsis ATH1 array was done in the LNBio (Brazilian Biosciences National Laboratory, Campinas, Brazil) Microarray Facility (LMA). Robust Multi‐array Average (RMA) normalization was performed using Expression Console™ (Affymetrix) and statistical data analysis was performed using the affylmGUI R package (R Core Team, 2018). A *P*‐value < 0.05 was used as the cutoff for the selection of differentially expressed genes and expression level between genotypes. Overlaps with previously available transcriptome data were carried out using the webtool Venn Diagram (http://bioinformatics.psb.ugent.be/webtools/Venn/), and statistical significance of overlaps were estimated using a web‐based software designed by Jim Lund (University of Kentucky), with statistical significance quantified using a hypergeometric test (Kim *et al*., 2001; http://nemates.org/MA/progs/overlap_stats.cgi). The heatmap in Fig. 1(f) was obtained using multiexperiment viewer (Saeed *et al*., 2003) and over‐representation of biological pathways were analyzed using mapman (Thimm *et al*., 2004). Microarray data are available at GEO (accession number GSE119175).

### ChIP

**Fig. 2:**
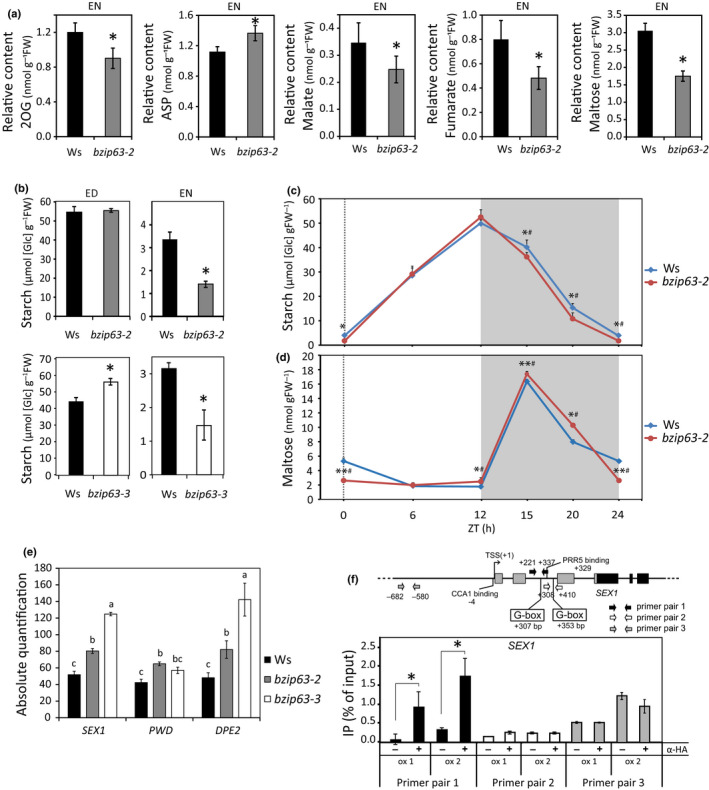
*bZIP63* modulates starch degradation. (a) Relative metabolite content at the end of the night (EN; ZT = 24 h) in leaves of *bzip63‐2* as compared to Wassilewskija (Ws). The concentrations of amino acids, organic acids and sugar were determined by gas chromatography time‐of‐flight mass spectroscopy (GC‐TOF‐MS). The average amount of metabolites was measured with five biological replicates, and each replicate consisted of leaves collected from six 25‐d‐old plants (two‐tailed Student’s *t*‐test; *n* = 30; *, *P* < 0.05). (b) Starch amounts at the end of the day (ED; ZT = 12 h) and EN (ZT = 24 h) in leaves of *bzip63‐2* and *bzip63‐3* mutants as compared to Ws. The average amount of starch for each time point were measured from two independent experiments, with five biological replicates, and each replicate consisted of leaves collected from six 30‐d‐old plants (two‐tailed Student’s *t*‐test; *n* = 60; *, *P* < 0.05; **, *P* < 0.01). Time‐course analysis of (c) starch and (d) maltose amounts determined by high‐performance liquid chromatography (HPLC) in *bzip63‐2* and Ws leaves through the diel cycle (12 h : 12 h, light : dark photoperiod). The average amount of starch and maltose for each time point were measured from two independent experiments, with five biological replicates, and each replicate consisted of leaves collected from six 30‐d‐old plants (two‐tailed Student’s *t*‐test; *n* = 60 for starch and 30 for maltose; *, *P* < 0.05; **, *P* < 0.01; and Wilcoxon’s test ^#^, *P* < 0.05). (e) Absolute transcript levels of starch degradation‐related genes *STARCH EXCESS1* (*SEX1*), *PHOSPHOGLUCAN*, *WATER DIKINASE* (*PWD*) and *DISPROPORTIONATING ENZYME 2* (*DPE2*), are higher in *bzip63‐2* and *bzip63‐3* at the end of the night (EN). Data were obtained from three independent experiments, with three biological replicates, and each replicate consisted of four youngest leaves and the shoot apical meristems collected from five 25‐d‐old plants, differences were tested performing ANOVA and the different letters indicate groups that are significantly different (Tukey’s honestly significant difference (HSD) test, *P* < 0.05). (f) Enrichment of specific regions of *SEX1* 5´sequences by chromation immunoprecipitation (ChIP) in *HA‐bZIP63*‐ox1 and *HA‐bZIP63*‐ox2 transgenic lines. bZIP63 binding to *SEX1* cis regulatory sequences was assessed by ChIP using anti‐HA antibody followed by quantitative PCR. Scheme of *SEX1* gene structure, depicting the binding sites of the circadian clock transcriptional regulators CCA1 and PRR5, as well as the bZIP63 binding regions containing canonical G‐box motifs. Primer pair 1 amplifies a 5′ region containing the closer G‐box motif relative to the transcription start site (TSS+1), whereas primer pair 2 amplifies a region containing the second closer G‐box motif relative to TSS+1, and the primer pair 3 amplifies a region without G‐box (−) indicates mock and (+) indicates immunoprecipitated samples. Data were obtained from four independent experiments for *HA‐bZIP63*‐ox1 and seven for *HA‐bZIP63*‐ox2 (Student’s *t*‐test; *, *P* < 0.05) for primer pairs 1 and 2, and one experiment for primer pair 3. Values are means, and error bars indicate standard deviation.

Samples were vacuum‐infiltrated with 1% formaldehyde solution in water and incubated at room temperature for 20 min to allow the crosslink of DNA‐protein complexes. After cross‐linking, samples were ground in liquid N_2_ using mortar and pestle. Extraction of DNA–protein complexes was performed as described previously (Gendrel *et al*., 2002) and resuspended in 300 μl nuclear lysis buffer (50 mM Tris‐HCL, pH 8; 10 mM EDTA; 1% SDS; 1× Pierce protease inhibitor cocktail #88265 (cat. no. A32963; Thermo Fisher Scientific). Re‐suspended DNA‐protein complexes were sonicated in a Bioruptor^®^ sonication system (Diagenode, Denville, NJ, USA) using the program, nine cycles of 30 min ON/1 min OFF with high potency, to achieve DNA fragments ranging from 75 to 500 bp, which was verified by agarose gel electrophoresis. DNA‐protein complexes were isolated using EpiQuik™ Plant ChIP kit (cat. no. P‐2014‐48; Epigentek Group Inc., Farmingdale, NY, USA) following the manufacturer’s instructions. Monoclonal anti‐HA antibody (cat. no. sc‐7392 C1313; Santa Cruz Biotechnology, TX, USA) combined with the Plant ChIP kit were used to capture HA‐tagged bZIP63‐DNA complexes. qPCR target enrichment analyses were performed using Platinum SYBR green (cat. no. 11733‐038; Invitrogen) run on a 7500 Applied Biosystems Fast Real‐time PCR System. Two transgenic lines overexpressing HA‐tagged bZIP63, *HA‐bZIP63*‐ox1 and *HA‐bZIP63*‐ox2 (Fig. 2f; Frank *et al*., 2018), were used for ChIP‐qPCR experiments. Overexpressor lines have been used previously for ChIP (e.g. Portolés & Más, 2010; Zheng *et al*., 2015; Frank *et al*., 2018); the two overexpressor lines accumulate approximately three‐fold more *bZIP63* transcript than the wild‐type (WT) at EN (Frank *et al*., 2018). Data were normalized using cycle threshold (Ct) derived from the comparison of the Ct of the anti‐HA antibody IP samples (adjusted relative to Ct of input DNA) and the Ct of mock IP (adjusted relative to Ct of the input DNA); Student’s *t‐*tests compared between the control and the anti‐HA treated samples as described previously (Haring *et al*., 2007).

### Phenotypic analysis

The leaf and rosette area (cm^2^) were measured using Leaf Area Meter 3100 (Li‐Cor Inc., Lincoln, NE, USA) or Phenoscope platform. For FW and DW measurements, rosettes were harvested, and the FW measured immediately. Afterwards, the same samples were dried in an oven at 70°C, and weighing was performed every 24 h until the samples reached a constant DW. Thirty plants were used for each genotype, discarding the three upper and lower outliers for each genotype. The epidermal cell area and the number of the rosette leaves were performed in an Eclipse 80i light microscope (Nikon, Tokyo, Japan). Samples were fixed using methanol, then cleared using lactic acid and mounted for analysis. The number of epidermal cells was obtained by counting the cells inside an area taken from 10 different positions across the leaf, corresponding to *c*. 10% of the total leaf area (leaves 1 and 3), and the total leaf area were measured, and from these values, the number of cells in the leaf epidermis was extrapolated. The Kolmogorov–Smirnov test was performed to ensure that the data were normally distributed and then Student’s *t*‐test was performed to identify significant differences between the *bzip63‐2* and Ws (*P* < 0.01).

### Extraction and quantification of starch

Starch was determined in the insoluble material after ethanolic extraction of soluble sugars, followed by enzymatic digestion with α‐amyloglucosidase and α‐amylase (Hendriks *et al*., 2003). Whole rosettes from six 30‐d‐old plants were used as a single sample. Samples were harvested immediately frozen in liquid N_2_, ground to a fine powder, and 20 mg FW measured and kept at −80°C until analysis.

### Metabolite profiling analysis

Forty milligrams of the grounded plant tissue were used for MTBE : methanol : water 3 : 1 : 1 (v/v/v) extraction, as described previously (Giavalisco *et al*., 2011). The 150 μl of the organic phase was dried and derivatized according to (Roessner *et al*., 2001). Then 1 μl derivatized samples were analyzed on a Combi‐PAL autosampler (Agilent Technologies) coupled to an Agilent 7890 gas chromatograph (GC) coupled to a Leco Pegasus 2 time‐of‐flight mass spectrometer (TOF‐MS) (LECO, St Joseph, MI, USA). Chromatograms were exported from Leco chromaTOF software (v.3.25) to R software. Peak detection, retention time alignment, and library matching were performed using R/targetsearch (Cuadros‐Inostroza *et al*., 2009). Metabolites were quantified by the peak intensity of a selective mass. Metabolite intensities were normalized by the FW, followed by the sum of total ion count. Each metabolite value was further normalized by the median of this given metabolite in all measured samples. For maltose, in addition to quantification by the method described above, another method based on high‐pressure anion‐exchange liquid chromatography (HPLC) separation was used as described in (Martins *et al*., 2013; Fig. 2d). The whole rosettes from six 30‐d‐old plants were used as a single sample. Samples were harvested and immediately frozen in liquid N_2_, and 100 mg grounded tissue was kept at −80°C until analysis.

### Statistical analyses

All statistical tests, *n* number, the measure of the means and the error bars are described in figure legends when appropriate. For comparison between the two groups, two‐tailed Student’s *t*‐test and Wilcoxon’s test were used. For multiple comparison statistical tests ANOVA followed by the Tukey’s honestly significant difference (HSD) test were performed using genes (Cruz, 2013). A hypergeometric test (Kim *et al*., 2001) was used to estimate the statistical significance of the overlap between transcriptomes. Analyses were considered significant at: *, *P* < 0.05; **, *P* < 0.01. Circadian oscillation parameters were calculated from two 24 h cycles under LL, excluding the first 24 h of data. The period was estimated using COSOPT and JTK_CYCLE, the significance thresholds were set to an adjusted *P* < 0.05 for JTK_CYCLE and a pMMC‐β < 0.05 for COSOPT (Hughes *et al*., 2010; Yang & Su, 2010). The phase and amplitude were estimated using meta cycle 2D with *P* < 0.05.

## Results

### *bZIP63* mutants display a photoperiod‐dependent growth impairment

*bZIP63* mediates energy stress responses triggered by SnRK1 (Baena‐González *et al*., 2007; Mair *et al*., 2015; Dröge‐Laser & Weiste, 2018), which suggests that *bZIP63* has an important role in energy management. Thus, we hypothesized that disruption of *bZIP63* expression could affect plant growth, especially when environmental conditions restrain photosynthesis or respiration, therefore limiting carbohydrate and ATP production. To verify this hypothesis, we performed a comparative analysis of the growth and development of *bZIP63* mutants and their respective WT, the Ws ecotype. We found that 30‐d‐old plants of the T‐DNA insertion mutants *bzip63‐2* and *bzip63‐3* had a rosette area nearly 25% smaller than Ws when grown under equinoctial conditions, but not under constant free‐running conditions (i.e. LL; Fig. 1a), and that the reduction of rosette size in the mutant was correlated with smaller leaf and epidermal cell area (Fig. 1b).

Because *bZIP63* is involved in the adjustment to an energy deficit, we reasoned that changes in photoperiod, and hence C availability through the day (Lu *et al*., 2005; Haydon *et al*., 2013; Dröge‐Laser & Weiste, 2018), could have an impact on the growth of *bZIP63* mutants. Indeed, the most contrasting rosette size shown by *bzip63‐2* and Ws was observed on plants grown in SD conditions, where *bzip63‐2* leaf and rosette area and DW were reduced by 30% and 46%, respectively (Figs 1c,d, S2b; Fig. S2a shows growth in 8 h : 16 h, light : dark photoperiod). In LD conditions, *bzip63‐2* leaf and rosette area and DW were reduced by only 17% and 30%, respectively, compared to Ws (Figs 1c,d, S2b). When plants were grown under LL conditions, the rosette area and weight of *bzip63‐2*, *bzip63‐3* and Ws were indistinguishable (Figs 1a,c,d). Altogether, the phenotypic data suggest that changes in photoperiod and the resulting alterations of C supply affect the impact of *bZIP63* on plant growth performance.

### *bZIP63* binds to energy deficit‐responsive genes

In order to obtain clues about the underlying reason of the growth impairment in the *bZIP63* mutant, we performed a comparative gene expression analysis of young leaves of *bzip63‐2* and Ws grown under SD and harvested at EN (= ZT 24), immediately before the onset of the light, to maximize the discovery of genes misregulated in *bzip63‐2* (Fig. S1e). The resulting gene expression profiles revealed 230 upregulated and 118 downregulated genes in *bzip63‐2* (Table S1). Among the downregulated genes in *bzip63‐2*, there was a 23% (*P* < 1.1e‐22) overlap with the genes induced by KIN10 overexpression, the SnRK1 catalytic subunit (Baena‐González *et al*., 2007; Table S1; Fig. 1e), which is consistent with the role of *bZIP63* in mediating KIN10‐induced transcriptional changes (Baena‐González *et al*., 2007; Mair *et al*., 2015). Noticeably, *ASPARAGINE SYNTHASE 1* (*ASN1*), *DARK INDUCIBLE 10* (*DIN10*), *BETA‐AMYLASE 9* (*BAM9*), *At2g30600* and *KISS ME DEADLY 4* (*KMD4/At3g59940*), that are induced by KIN10 and by C starvation (Contento *et al*., 2004; Gibon *et al*., 2004; Baena‐González *et al*., 2007; Usadel *et al*., 2008; Cookson *et al*., 2016), and can therefore be considered as low C/energy marker genes, were repressed in *bzip63‐2* (Fig. 1f). *ASN1*, *BAM9* and *At2g30600* were induced in two transgenic lines overexpressing *HA:VP16:bZIP63* fusion (*HA‐bZIP63*‐ox1 and *HA‐bZIP63*‐ox2; Table S2). Target enrichment analysis using ChIP followed by qPCR showed that bZIP63 binds to the 5′‐sequences of these genes *in vivo* (Fig. S3a–c), suggesting that they are direct targets of bZIP63. These results corroborate the role of bZIP63 as a key transcription factor acting downstream of KIN10 to reprogram transcription in response to low energy stress (Baena‐González *et al*., 2007; Matiolli *et al*., 2011; Mair *et al*., 2015).

### *bZIP63* mutants show faster starch degradation during the night

A subset of 10 genes that are induced by KIN10 overexpression (*P* < 6.8e‐07; Baena‐González *et al*., 2007) and various conditions of C depletion (*P* < 3.3e‐07; Contento *et al*., 2004; Gibon *et al*., 2004; Usadel *et al*., 2008; Cookson *et al*., 2016; Fig. 1e), and therefore also can be considered as low C/energy marker genes, were induced in *bzip63‐2* (Fig. 1e,f). This result suggests that the management of energy supply is impaired in *bzip63‐2*. To further evaluate this possibility, we performed a comparative primary metabolic profiling analysis between *bzip63‐2* and WT at EN using GC‐TOF‐MS (Table S3). We found that *bzip63‐2* had a reduction of the concentrations of 2‐oxoglutarate (2OG) (Fig. 2a), an intermediate of the TCA cycle and an important C‐skeleton for glutamine synthesis by glutamate synthase from glutamate in N assimilation. 2OG therefore is considered to integrate C and N metabolism (Nunes‐Nesi *et al*., 2010). The reduced 2OG content by contrast with the unchanged concentrations of glutamate and glutamine is consistent with the limiting C availability (Nunes‐Nesi *et al*., 2010; Fig. 2a; Table S3). Furthermore, larger amounts of aspartate (ASP) in the mutant than in the WT (Fig. 2a), also may reflect a decrease in available sugars at the EN (Nunes‐Nesi *et al*., 2010). Additionally, malate and fumarate, which can fuel respiration to provide energy when starch reserves are exhausted (Zell *et al*., 2010), also were decreased in *bzip63‐2* (Fig. 2a), further suggesting a superimposed shortage of C/energy. Finally, maltose, the major product of starch breakdown, was diminished in the mutant (Fig. 2a), suggesting faster exhaustion of starch reserves. Indeed, *bzip63‐2* and *bzip63‐3* had 50% less starch at EN compared to Ws (Fig. 2b). The amount of starch accumulated in these mutants was similar (*bzip63‐2*, Fig. 2b) or slightly higher (*bzip63‐3*, Fig. 2b) at the end of the day (ED). These results suggest that *bzip63‐2* and *bzip63‐3* degrade starch faster during the night. To get a more detailed picture of how starch metabolism is affected by bZIP63, we performed a time‐course analysis of starch and maltose, measured by HPLC, in *bzip63‐2* during a 24 h diel cycle (equinoctial conditions, Fig. 2c,d; SD, Fig. S4a,b). The differences in starch content between *bzip63‐2* and Ws were significant as early as 3 h after the beginning of the night (ZT15, Fig. 2c; ZT13, Fig. S4a), suggesting that *bZIP63* mutation affects starch degradation since ED. The amounts of maltose in *bzip63‐2* were higher than those observed for Ws at the beginning of the night (i.e. ZT15 and ZT20; Fig. 2d), which correlates with the faster starch breakdown found in this mutant compared to Ws (Fig. 2c). The lower concentrations of maltose in *bzip63‐2* at EN measured by both HPLC and GC‐TOF‐MS most likely results from a reduction of its production as a consequence of premature exhaustion of starch reserves (Fig. 2a,d).

We found that the transcript levels of two starch phosphorylating enzymes, namely *α‐GLUCAN*, *WATER DIKINASE1/STARCH EXCESS1* (*GWD1*/*SEX1*) and *PHOSPHOGLUCAN*, *WATER DIKINASE* (*PWD*), which are required for starch degradation (Mahlow *et al*., 2014), were higher in both *bzip63‐2* and *bzip63‐3* mutants at EN (Figs 2e, S4c; Tables S1, S2, S4). In addition, the cytosolic maltotriose‐metabolizing enzyme *DISPROPORTIONATING ENZYME 2* (*DPE2*), an essential component of the pathway that releases glucose from maltose (an intermediate of starch breakdown; Fettke *et al*., 2006), also was induced in both *bzip63‐2* and *bzip63‐3* (Figs 2e, S4c; Tables S1,S2). Subsequent ChIP‐qPCR analysis showed that VP16:HA‐tagged bZIP63 binds to the 5′ sequence of *SEX1* (Fig. 2f), suggesting that this gene is a direct target of bZIP63. These data establish a positive correlation between higher *SEX1*, *PWD* and *DPE2* transcript levels and faster starch degradation in the mutants. Altogether, the results indicate that the faster starch degradation in the *bZIP63* mutants and the consequent early depletion of starch at the EN (Figs 2b,c, S4) led to low energy stress, which may explain – at least in part – the growth defect of these mutants. This hypothesis is consistent with the notion that C/energy starvation towards dawn impairs growth (Smith & Stitt, 2007; Graf *et al*., 2010; Apelt *et al*., 2017).

### The circadian clock and C/energy status interact to regulate *bZIP63* diel expression pattern

**Fig. 3:**
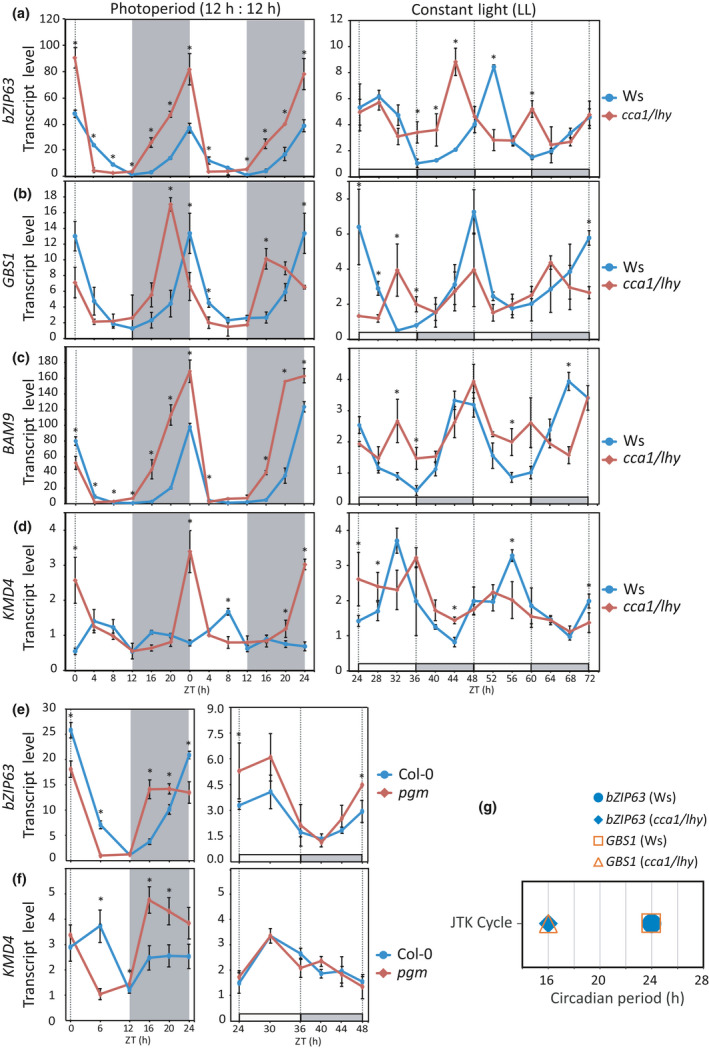
The circadian clock and carbon (C)/energy status control *bZIP63* oscillatory pattern. (a) Circadian oscillation of *bZIP63* transcript levels follows the shorter period of the double mutant *cca1/lhy* under free‐running conditions (LL), but does not show the expected phase advance under photoperiod (CCA1, CIRCADIAN CLOCK ASSOCIATED 1; LHY, Late Elongated Hypocotyl). (b) Transcripts levels of *GBS1*, which is a gene under circadian clock regulation, follows the shorter period of *cca1/lhy* in LL and shows a phase advance in photoperiod. (c) The bZIP63 direct target *BAM9* presents transcript level oscillation following the shorter period of *cca1/lhy* in LL, but does not have the phase advance under photoperiod. (d) Under photoperiod, the C/energy starvation marker *KMD4* is induced during the last part of the night in *cca1/lhy*. (e) *bZIP63* transcript oscillation pattern is altered in the starch synthesis deficient mutant *pgm* under photoperiod, showing a rapid increase in the first hours of the night, which is consistent with the decrease of sugar concentrations at this time (Gibon *et al*., 2004). By contrast, transcript levels of *bZIP63* were similar between *pgm* and Col‐0 in the early subjective night in LL. (f) The transcript level of the starvation marker *KMD4* is increased at early night in *pgm*, whereas this is not observed in the early subjective night in LL. (g) Transcript levels of *bZIP63* and *GBS1* follow the shorter period of the double mutant *cca1/lhy* under LL. In the plotted graphs of photoperiod, white or gray backgrounds represents light or dark period, respectively. In graphs of LL, the *x*‐axis represents the time elapsed after releasing the plants into LL. The subjective day (ZT 24–36, 48–60) and subjective night (ZT 36–48, 60–72) are identified by white and gray bars on the *x*‐axis, respectively. The data were obtained from two independent experiments, with three biological replicates at each time point, and each replicate consisted of the whole rosette collected from three plants (two‐tailed Student’s *t*‐test; *n* = 9; *, *P* < 0.05). Values are means, and error bars indicate standard deviation.

The circadian clock is crucial to define the rate of starch breakdown compatible with a regular C supply during the diel cycle (Lu *et al*., 2005; Graf *et al*., 2010; Scialdone *et al*., 2013; Pokhilko *et al*., 2014; Seki *et al*., 2017; Seaton *et al*., 2018). We demonstrated that *bZIP63* is essential for circadian clock entrainment by sugars, transducing this metabolic signal into the core oscillator through the transcriptional regulation of the clock component *PSEUDO‐RESPONSE REGULATOR* (*PRR*)*7* (Frank *et al*., 2018). In addition, previous reports showed that the circadian clock transcriptional repressors CIRCADIAN CLOCK ASSOCIATED 1 (CCA1), PRR5 and PRR7 bind to the *bZIP63* promoter (Nagel *et al*., 2015; Liu *et al*., 2016), and that *bZIP63* expression is higher in both the *prr579* triple mutant (Liu *et al*., 2016) and the *cca1/lhy* double mutant (Graf *et al*., 2017) when compared to the respective WT accessions, suggesting that *bZIP63* expression is under circadian clock control. Thus, we investigated in more detail the reciprocal regulation between *bZIP63* and the circadian clock, aiming to shed light on how starch degradation and energy stress responses are regulated in a timely manner. We first verified whether *bZIP63* expression is regulated by the circadian clock. Indeed, *bZIP63* transcript level oscillates in free‐running conditions in both Ws and the circadian clock mutant *cca1/lhy* (Graf *et al*., 2010), closely matching the Ws period (*c.* 24 h) and the shorter period (*c.* 17 h) of *cca1/lhy* (Fig. 3a,g; Table S5). As expected, the expression of the circadian clock‐regulated gene *GRANULE BOUND STARCH SYNTHASE 1* (*GBS1*; Graf *et al*., 2010) showed a shorter oscillation period in free‐running conditions and a phase advance in diel conditions in the *cca1/lhy* mutant as compared to Ws (Fig. 3b,g; Table S5). These results demonstrate that *bZIP63* is under circadian clock control. The similarity of *bZIP63* and *BAM9* expression profiles (Fig. 3a,c) is compatible with *BAM9* being a target of bZIP63 (Figs 1f, S3; Table S2).

Interestingly, in the *cca1/lhy* mutant grown in diel conditions, *bZIP63* transcript level – by contrast to that of *GBS1* – peaked at dawn as in Ws (Fig. 3a,b). Moreover, the *bZIP63* transcript level oscillation showed a higher amplitude in *cca1/lhy* than in Ws (ZT16; Fig. 3a). We reasoned that, as *bZIP63* expression is regulated by C/energy levels (Baena‐González *et al*., 2007; Matiolli *et al*., 2011; Mair *et al*., 2015), its oscillation pattern in *cca1/lhy* grown in light : dark cycles could be due to C/energy starvation towards EN as a consequence of the premature exhaustion of starch reserves (Graf *et al*., 2010). Energy starvation in *cca1/lhy* was supported by the induction of the C/energy starvation marker gene *KMD4* (Graf *et al*., 2010) in the last hours of the night under photoperiodic growth conditions (Fig. 3d). In addition, the phase advance of *bZIP63* expression in the starchless mutant *pgm* under photoperiod (Fig. 3e) reinforces the notion that *bZIP63* expression is regulated by C/energy levels and the circadian clock. The *pgm* mutant does not accumulate any significant amount of starch and, therefore, experiences reduced C and energy availability early in the night (Gibon *et al*., 2004), as demonstrated by the stronger induction of *KMD4* in this mutant (Fig. 3f). Under LL conditions, in which C availability is not limiting, no differences in *bZIP63* and *KMD4* expression were found between Col‐0 and *pgm* (Fig. 3e,f). These results provide strong support for the notion that *bZIP63* transcript oscillation pattern (i.e. phase and amplitude) is set by the energy levels and the circadian clock.

### Interaction between *bZIP63* and circadian clock regulates starch degradation‐related genes and energy deficit‐responsive genes

We observed that 60% of the misregulated genes in *bzip63‐2* mutant oscillate under LL conditions (Table S1), which represents a two‐fold enrichment compared to circadian clock‐controlled oscillating genes in Arabidopsis (Covington *et al*., 2008; Harmer, 2009). These findings suggest that the interaction between *bZIP63* and the circadian clock (Fig. 3; Frank *et al*., 2018) impacts the bZIP63 transcriptional output. Thus, we investigated the importance of this interaction on the expression pattern of genes related to starch degradation and energy deficit responses (Tables S1, S2).

**Fig. 4:**
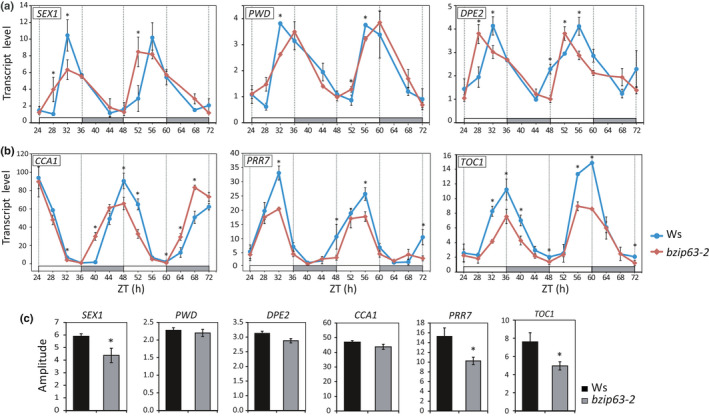
bZIP63 modulates the oscillatory pattern of circadian clock and starch degradation related genes. Transcript oscillatory pattern of starch degradation related genes (a), and circadian clock genes (b), are altered in *bzip63‐2* as compared to Ws. 30‐d‐old plants were entrained under 12 h : 12 h, light : dark photoperiod and released into free‐running conditions (LL) for 3 d. The *x*‐axis represents the time elapsed after release of the plants into LL, where subjective day (ZT 24–36; 48–60) and subjective night (ZT 36–48; 60–72) are identified by white and gray bars on the *x*‐axis, respectively. Transcript oscillation amplitude of starch degradation‐related genes *STARCH EXCESS1* (*SEX1*), *PHOSPHOGLUCAN*, *WATER DIKINASE* (*PWD*) and *DISPROPORTIONATING ENZYME 2* (*DPE2*) and the circadian clock genes *CIRCADIAN CLOCK ASSOCIATED 1* (*CCA1*), *PSEUDO‐RESPONSE REGULATOR 7* (*PRR7*) and *TIMING OF CAB EXPRESSION 1* (*TOC1*) under LL (c). The data were obtained from two independent experiments, with three biological replicates in each time point, and each replicate consisted of the whole rosette collected from three plants (two‐tailed Student’s *t*‐test; *n* = 9; *, *P* < 0.05). Values are means, and error bars indicate standard deviation.

We showed that the transcript levels of the starch degradation‐related genes *SEX1*, *PWD* and *DPE2* are regulated by bZIP63, as they are induced in *bzip63‐2* and *bzip63‐3* mutants at EN (Fig. 2e; Table S2). Because the expression of these genes also is known to be regulated by the circadian clock (Ni *et al*., 2009; Edwards *et al*., 2010; Seo *et al*., 2012), we would expect their circadian oscillation pattern to be altered in *bZIP63* mutants. Thus, we performed a comparative analysis of *SEX1*, *PWD* and *DPE2* transcript level oscillation in *bzip63‐2* and Ws leaves from plants released into LL conditions rather than under SD or LD cycles, to circumvent possible confounding effects related to energy fluctuations in diel conditions (Fig. 4a; Table S2).

All three genes exhibited different oscillation patterns in *bzip63‐2* compared to Ws (Fig. 4a), with lower oscillation amplitude for *SEX1* (Fig. 4c; Table S5), and phase advance and delay for *DPE2* and *PWD* transcripts, respectively, in *bzip63‐2* (Fig. 4a; Table S5). No significant differences in *SEX1* and *PWD* mRNA levels between WT and *bzip63*‐2 were detected at the subjective EN under LL conditions (Fig. 4a) as would be expected from the data under diel growth conditions (Fig. 2e). This discrepancy could be related to differences of unknown regulatory features between diel and free‐running growth conditions, such as those deriving from changes in the pattern of C flow.

Expression of *SEX1* is deregulated in *bZIP63* mutants, and bZIP63 binds to *SEX1* 5′‐ sequences (Fig. 2e,f). Moreover, the circadian clock component PRR5 was shown to bind next to the region bounded by bZIP63 in *SEX1* 5′‐ sequences (Liu *et al*., 2016; Fig. 2f). In addition, CCA1 also was found to bind to the *SEX1* promoter (Nagel *et al*., 2015; Fig. 2f). Therefore, regulation of *SEX1* expression could be partly driven by direct targeting of bZIP63 and components of the circadian oscillator to its 5´sequences (Fig. 2f). Because we were unable to show that bZIP63 binds to *PWD* and *DPE2* promoters, it is possible that bZIP63 indirectly regulated the expression of these two genes by influencing the circadian oscillator through regulation of *PRR7* expression (Frank *et al*., 2018; Fig. 4b,c). Indeed, under LL conditions, we found that the phase of *CCA1* transcript oscillation was advanced in the *bzip63‐2* mutant (Fig. 4b), which is consistent with downregulation of its transcriptional repressor *PRR7* in *bzip63‐2* (Frank *et al*., 2018; Fig. 4b,c; Table S2). *PRR7* and *TIMING OF CAB EXPRESSION 1* (*TOC1*), which are involved, respectively, in the morning and central loops of the core clock oscillator, oscillate with lower amplitude in *bzip63‐2* (Fig. 4b,c). All of these changes indicate that *bZIP63* could modulate the circadian expression of *PWD* and *DPE2* through the adjustment of the circadian clock. Therefore, these results suggest that bZIP63 activity can affect the circadian expression of *SEX1*, *PWD* and *DPE2* by both direct (e.g. by binding to *SEX1* 5′‐sequences) and indirect mechanisms by adjusting the circadian oscillator and – consequently – its gene expression output.

We found that 28 downregulated genes in *bzip63‐2* (23%, *P* < 1.1e‐22) overlapped with genes induced by KIN10 overexpression (i.e. energy stress responsive genes, Fig. 1e; Table S1). Among these genes, 11 (41%, *P* < 1.1e‐19) oscillate with the same phase as *bZIP63* (ZT 23) under entrainment conditions (Mockler *et al*., 2007; Fig. S5). This observation raises the possibility that, in a similar way to the starch degradation‐related genes, the expression of a subset of KIN10‐induced energy‐stress responsive genes is controlled by the interaction between *bZIP63* and the circadian clock. This assumption was supported by the observation that the upstream regions of two of these KIN10‐induced energy‐stress responsive genes, namely *BAM9* (Figs 1f, 3c) and *At2g30600* (Fig. 1f) are bound by bZIP63 (Fig. S3b,c) in a region overlapping the sequences bound by both PRR5 and PRR7, and PRR5, respectively (Liu *et al*., 2016). This dual regulatory scheme of bZIP63 and the circadian clock could explain the higher amplitude of the rhythmic oscillation of *BAM9* transcript levels in *cca1/lhy* under photoperiod (Fig. 3c) probably as a consequence of energy limitation resulting from premature exhaustion of starch (Graf *et al*., 2010). Altogether, the evidence suggests that *bZIP63* misregulation has a broad impact on the circadian oscillation of core oscillator genes, which modulates the circadian clock output, such as starch metabolism and responses to low energy signals.

## Discussion

During daylight, plants harvest energy from sunlight to synthesize soluble sugars to support metabolism and growth. A fraction of the photoassimilates is saved in the form of storage carbohydrates, such as the leaf transitory starch, to ensure energy supply during the night. Appropriate management of energy resources is crucial to promote growth and ensure reproduction, thus increasing fitness. We show here that the transcription factor bZIP63, a key phosphorylation target of SUCROSE non‐fermenting RELATED KINASE1 (SnRK1) (Baena‐González *et al*., 2007; Mair *et al*., 2015), is essential for optimal growth, probably through its involvement in managing energy resources. By comparison with the wild‐type (WT), *bzip63‐2* and *bzip63‐3* mutants exhibited faster night‐time starch degradation, which was correlated with lower amounts of metabolites related to carbon (C) status (Fig. 2a; Table S3), indicating a situation of energy deficit at the end of the night (EN). Reduction of available energy in *bZIP63* mutants at EN also is supported by the induction of the expression of C/energy‐starvation related genes such as *Beta‐Galactosidase 4* and Dormancy‐associated protein 1 (Fig. 1e,f; Table S2). Our data are in agreement with previous results showing that even a small reduction in the starch content after an artificial extension of the night period resulted in a significant reduction of carbohydrates and organic acids and an induction of energy deficit marker genes (Gibon *et al*., 2004; Usadel *et al*., 2008; Graf *et al*., 2010). Thus, in a similar way to other mutants impaired in starch metabolism, such as *phosphoglucomutase* (*pgm*), *STARCH EXCESS1* (*sex1‐1*), *BETA‐AMYLASE 3* (*bam3*) and *bam4* (Fulton *et al*., 2008; Paparelli *et al*., 2013), or the circadian clock mutant *cca1/lhy* (Graf *et al*., 2010) (CCA1, Circadian Clock Associated 1; LHY, Late Elongated Hypocotyl), the reduced growth phenotype observed in *bzip63‐2* and *bzip63‐3* possibly is due to reduced C supply, and the resulting energy deficit, towards EN (Graf *et al*., 2010; Kölling *et al*., 2015; Mair *et al*., 2015). This conclusion is supported by the equalization of WT and *bZIP63* mutants growth under constant light, which provides a stable photosynthate supply throughout the day. A similar phenomenon has been described for the starch metabolism mutants *pgm* and *sex1‐1* (Izumi *et al*., 2013). The involvement of *bZIP63* in the growth optimization seems to be more critical under short day (SD) conditions (Fig. 1c,d), where less energy is available in a 24 h period (Sulpice *et al*., 2014), partly because less starch is accumulated at the end of the day (ED) in SD conditions in comparison to long day (LD) conditions (Fig. S6a). In addition, the small amount of starch at EN of SDs in *bzip63‐2* (Fig. S6a) most likely resulted in a stronger energy starvation, which is supported by the greater accumulation of transcripts of the energy deficit marker *ASN1* (Fig. S6b). Thus, accentuated growth impairment of *bzip63‐2* in SD conditions (Figs 1c,d, S2a,b) is likely a consequence of a stronger energy deficit towards the EN. These results indicate that *bZIP63* is involved in the regulation of starch degradation, which supports the proposed role of *bZIP63* in C and energy metabolism management (Baena‐González *et al*., 2007; Mair *et al*., 2015). The reduced *bzip63‐2* mutant leaf cell size (Fig. 1b) can partly explain the smaller leaf area of this mutant, and is correlated with the downregulation of cell wall remodeling *XYLOGLUCAN ENDOTRANSGLUCOSYLASE/HYDROLASE* genes (Table S1). One of them, *XTH15,* whose expression was correlated with cell elongation (Pedmale *et al*., 2016), is regulated by sugar concentrations (Matiolli *et al*., 2011) and was found to be involved in cell wall remodeling (Hayashi & Kaida, 2011). *XTH15* also was found to be regulated by bZIP63 (Fig. S7a,b), and also by the circadian oscillator through the circadian clock component PRR5 (Liu *et al*., 2016). Hence, these results suggest that *bZIP63* may directly participate in the regulation of cell expansion.

Starch degradation is controlled by the circadian clock to maintain a constant sugar supply required for growth during the night (Smith & Stitt, 2007; Graf *et al*., 2010; Sulpice *et al*., 2014; Flis *et al*., 2016). The faster starch degradation observed in *bzip63‐2* and *bzip63*‐*3* is correlated with the upregulation of genes encoding the starch degradation‐related enzymes *PHOSPHOGLUCAN*, *WATER DIKINASE* (*PWD*) and *DISPROPORTIONATING ENZYME 2* (*DPE2*) at EN but a direct causal relationship remains to be established. In any case, the diel fluctuation of *SEX1*, *PWD* and *DPE2* transcript levels possibly is regulated by the circadian clock through direct binding of CCA1 and PRR5 to their promoters (Nagel *et al*., 2015; Liu *et al*., 2016), and bZIP63 because the phase or amplitude of the oscillation pattern of these transcripts were changed in *bzip63‐2* under free‐running (LL) conditions (Fig. 4a,c; Table S5). *bZIP63* was shown to have a direct input on the circadian clock by participating in its entrainment by sugars (Frank *et al*., 2018). In turn, as shown here, the circadian clock feeds back to regulate *bZIP63* expression, which combined with C/energy levels, shapes the diel oscillation pattern of *bZIP63* transcript levels (Fig. 3a,e). Thus, *bZIP63* is part of a regulatory module that integrates C/energy and circadian clock signals to fine‐tune the circadian transcript level oscillation of the starch degradation‐related genes *PWD*, *SEX1* and *DPE2*. This regulation may be achieved by the direct regulation of target genes, as suggested by bZIP63 binding to a *SEX1* 5′ sequence containing a canonical bZIP G‐box binding site localized next to a PSEUDO‐RESPONSE REGULATOR 5 (PRR5) target region (Fig. 2f). Alternatively, bZIP63 may act indirectly, such as in the case of *PWD* and *DPE2*, through the regulation of the circadian oscillator in response to sugars (Frank *et al*., 2018; Figs 2e, 4a,c). The same regulatory rationale may also apply to 22 of 28 (78%) C/energy deficit responsive genes downregulated in *bzip63‐2* (i.e. induced by KIN10 overexpression; Baena‐González *et al*., 2007; Fig. 1e; Table S1), which were shown to be bound by at least one of the core oscillator genes *CCA1*, *PRR5*, *PRR7*, *PRR9* and *TIMING OF CAB EXPRESSION 1* (*TOC1*) (Nagel *et al*., 2015; Liu *et al*., 2016; Table S6). Indeed, we show that bZIP63 binding regions co‐localize with sequences bound by circadian clock components in the 5′ sequences of the energy stress marker genes *BAM9* and *At2g30600* (Edwards *et al*., 2010; Liu *et al*., 2016; Fig. S3b,c; Table S6). Based on these observations, we speculate that bZIP63, interacting with PRRs, is involved in defining the oscillation pattern of downstream genes as part of a transcriptional gating mechanism similar to the co‐occupancy of circadian clock‐regulated genes by PRRs and the *PHYTOCHROME INTERACTING FACTORS PIF3* and *PIF4* to mediate the photosensory pathway and thermoresponsive growth, respectively (Soy *et al*., 2016; Zhu *et al*., 2016; Martín *et al*., 2018).

bZIP63 also may have opposite regulatory activities because the expression of *SEX1* and stress‐related genes (e.g. *BAM9*) are up‐ and downregulated, respectively, in *bZIP63* mutants (Fig. 1e, 2e). This antagonistic regulatory feature may depend on the architecture of the promoter that bZIP63 binds to and consequently its interaction with other transcription factors. For instance, the circadian clock regulator CCA1‐HIKING EXPEDITION (CHE), one of the protein interactors of bZIP63 (Frank *et al*., 2018), can bind to the promoters of *ISOCHORISMATE SYNTHASE 1* (*ICS1)* and *CCA1*, acting as an activator and repressor, respectively (Pruneda‐Paz *et al*., 2009; Zheng *et al*., 2015). bZIP63 regulatory output also may be modulated by its interaction with a set of other transcription factors (i.e. TCP2, 4, 10 and 14, NAC066, AtMYB56, AtWOX13 and type B Response Regulators ARR18; Veerabagu *et al*., 2014; Trigg *et al*., 2017). Finally, bZIP63‐related transcriptional network also will be shaped by its pattern of heterodimerization with the S‐group bZIP1, 2 or 53, which to some extent involves its phosphorylation by SnRK1 (Ehlert *et al*., 2006; Mair *et al*., 2015; Pedrotti *et al*., 2018).

**Fig. 5:**
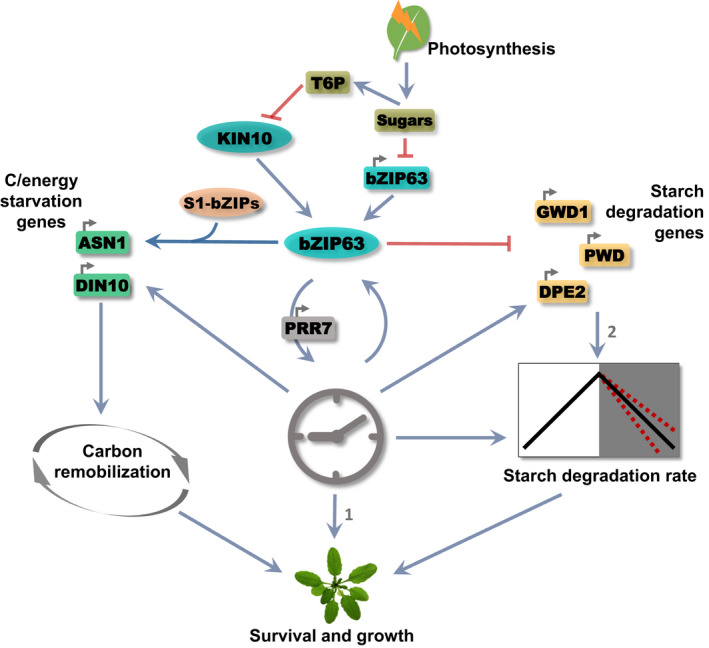
*bZIP63* regulates plant growth. The circadian clock and sugars deriving from photosynthesis set the *bZIP63* expression pattern. The regulation of energy starvation and starch degradation‐related genes, and the circadian clock gene *PSEUDO‐RESPONSE REGULATOR 7* (*PRR7*) expression by bZIP63 may depend upon its phosphorylation by KIN10, which induces the bZIP63/S1‐bZIPs network (Mair *et al*., 2015). KIN10 activity is inhibited by Trehalose‐6‐Phosphate (T6P), a proxy of sucrose concentrations (Frank *et al*., 2018; Zhai *et al*., 2018). The model proposes two possible modes by which *bZIP63* modulates growth: through carbon remobilization to cope with stress or by regulating *PRR7* expression to adjust the pace of the circadian clock in response to sugars which would affect the starch degradation rate. These processes are likely to be interlocked by the daily sugar and circadian clock dynamics: (1) circadian clock impacts growth (Dodd *et al*., 2005; Nozue *et al*., 2007; Farré, 2012); (2) the direct impact of *STARCH EXCESS1* (*SEX1*), *PHOSPHOGLUCAN*, *WATER DIKINASE* (*PWD*) and *DISPROPORTIONATING ENZYME 2* (*DPE2*) induction in bZIP63 mutants on the starch degradation rate is unknown. Blunt ended arrows indicate repression while arrows indicate activation.

We provide evidence that bZIP63 interacts with the circadian clock to establish the rate of starch degradation, which will ensure adequate C/energy supply throughout the diel cycle and, therefore, optimize growth performance. We suggest that the integration of C/energy levels and circadian clock‐related signals by bZIP63 makes it a player in acclimation responses to environmental‐induced changes in C/energy availability (Fig. 5).

## Author contributions

AJCV and CCM designed experiments, collected and analyzed all of the data; DWN performed ChIP‐qPCR and analyzed the data; MCMM and CC performed and analyzed metabolomics data and advised the interpretation; CTH analyzed transcript oscillation data and advised the interpretation; GTD and JGPV performed an experiment under diel condition; EG performed and analyzed the phenotypic experiment on Phenoscope platform; MV designed experiments, analyzed all of the data and obtained the funding; and AJCV, CCM, CC and MV wrote the manuscript. AJCV and CCM contributed equally to this work.

## Supporting information


**Fig. S1** Characterization of *bZIP63* mutants.
**Fig. S2** *bzip63‐2* mutant has a reduced leaf area and growth rate.
**Fig. S3** bZIP63 binds to energy stress‐responsive genes.
**Fig. S4** *bzip63‐2* starch degradation pattern under SD conditions.
**Fig. S5** Circadian phase of KIN10‐induced energy‐stress responsive genes.
**Fig. S6** Starch availability in SD vs 12 h : 12 h photoperiods.
**Fig. S7** bZIP63 binds to cell wall modification gene.

**Table S1** Up‐ and downregulated genes in *bzip63‐2* plants compared with the WT.
**Table S2** Genes related to circadian clock, starch degradation and energy stress deregulated in *bZIP63* mutants.
**Table S3** Relative metabolite content at EN in leaves of *bzip63‐2* as compared to Ws.
**Table S4** List of primers used for quantification of the mRNA levels and the immunoprecipitated sequences by qRT‐PCR.
**Table S5** Circadian parameters calculated from two 24‐h cycles under LL conditions.
**Table S6** Circadian oscillator binding in downregulated genes in *bzip63‐2* that are induced by KIN10 overexpression.
Please note: Wiley Blackwell are not responsible for the content or functionality of any Supporting Information supplied by the authors. Any queries (other than missing material) should be directed to the *New* *Phytologist* Central Office.
