# Supplementary material for: The sugar‐responsive circadian clock regulator bZIP63 modulates plant growth
Source: New Phytol. 2021 Jun 30;231(5):1875–89. doi: 10.1111/nph.17518 (PMC9292441; doi:10.1111/nph.17518)
Supplement: Supplementary file 1 — Fig. S1 Characterization of bZIP63 mutants. Fig. S2 bzip63‐2 mutant has a reduced leaf area and growth rate. Fig. S3 bZIP63 binds to energy stress‐responsive genes. Fig. S4 bzip63‐2 starch degradation pattern under SD conditions. Fig. S5 Circadian phase of KIN10‐induced energy‐stress responsive genes. Fig. S6 Starch availability in SD vs 12 h : 12 h photoperiods. Fig. S7 bZIP63 binds to cell wall modification gene. [file NPH-231-1875-s001.pdf]

***New Phytologist* Supporting Information**

Article title: **The sugar-responsive circadian clock regulator bZIP63 modulates plant growth**

Authors: Américo J. C. Viana[1,*], Cleverson C. Matiolli[1,*], David W. Newman[1], João G. P. Vieira[1], Gustavo T. Duarte[1], Marina C. M. Martins[2,5], Elodie Gilbault[3], Carlos T. Hotta[4], Camila Caldana[2,6], Michel Vincentz[1].

[1]Centro de Biologia Molecular e Engenharia Genética, Departamento de Biologia Vegetal, Instituto de Biologia, Universidade Estadual de Campinas, CEP 13083-875, CP 6010, Campinas, São Paulo, Brazil. [2]Brazilian Bioethanol Science and Technology Laboratory (CTBE/CNPEM), Rua Giuseppe Máximo Scolfaro 10000, 13083-970, Campinas, Brazil. Max-Planck Partner Group, Brazilian Bioethanol Science and Technology Laboratory (CTBE/CNPEM), Campinas, Brazil. [3]Institut Jean-Pierre Bourgin, INRA, AgroParisTech, CNRS, Université Paris-Saclay, 78000, Versailles, France. [4]Departamento de Bioquímica, Instituto de Química, Universidade de São Paulo, CEP 05508-000, São Paulo, SP, Brazil. [5]Laboratory of Plant Physiological Ecology, Department of Botany, Institute of Biosciences, University of São Paulo, CEP 05508-090, São Paulo, Brazil. [6]Max Planck Institute of Molecular Plant Physiology, Am Mühlenberg 1, 14476, Potsdam, Golm, Germany.

Author for correspondence: Michel Vincentz, Tel: +55 (19) 3521-1140

Email: mgavince@unicamp.br

[*]These authors contributed equally to this work.

Article acceptance date: 18 May 2021.

The following Supporting Information is available for this article:

Fig. S1 Characterization of *bZIP63* mutants.

Fig. S2 *bzip63-2* mutant has a reduced leaf area and growth rate.

Fig. S3 bZIP63 binds to energy stress responsive genes.

Fig. S4 *bzip63-2* starch degradation pattern under short days.

Fig. S5 Circadian phase of KIN10-induced energy-stress responsive genes.

Fig. S6 Starch availability in short day versus 12-12 photoperiods.

Fig. S7 bZIP63 binds to cell wall modification gene.

Table S1 Up and Down-regulated genes in *bzip63-2* plants compared with the WT.

Table S2 Genes related to circadian clock, starch degradation and energy stress deregulated in *bZIP63* mutants.

Table S3 Relative metabolite content at EN in leaves of *bzip63-2* as compared to Ws.

Table S4 List of primers used for quantification of the mRNA levels and the immunoprecipitated sequences by RT-qPCR.

Table S5 Circadian parameters calculated from two 24 h cycles under free-running conditions.

Table S6 Circadian oscillator binding in downregulated genes in *bzip63-2* that are induced by KIN10 overexpression.

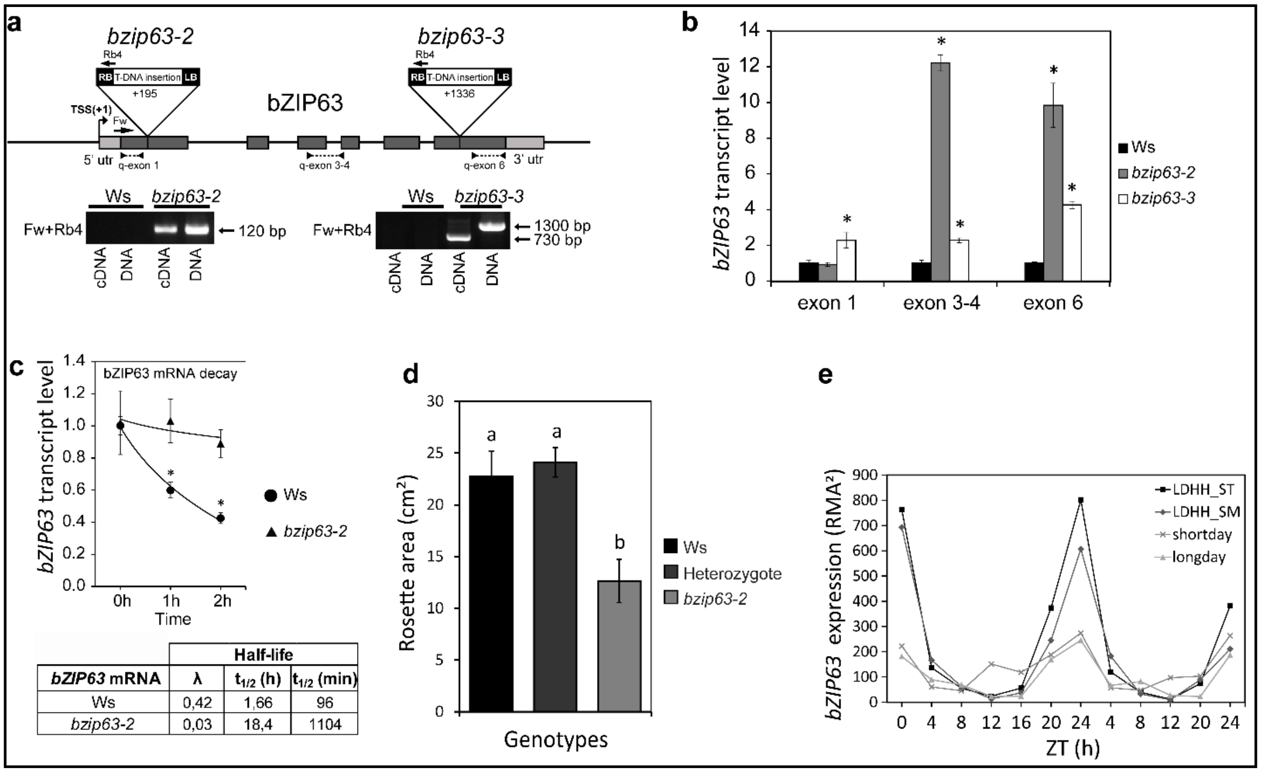

Fig. S1 Characterization of *bZIP63* mutants. (a) Schematic representation of *bZIP63* gene structure depicting the T-DNA insertion location in *bzip63-2* (FLAG_610A08) and *bzip63-3* (FLAG_532A10) mutants. T-DNA insertion and chimeric transcripts were verified by end-point PCR using genomic DNA and cDNA from wild type Ws and mutants *bzip63-2* and *bzip63-3*; arrows indicate end-point PCR primers (Fw: forward primer and Rb4: T-DNA right border primer; Table S4) and horizontal dashed lines indicate the positions of the RT-qPCR products shown in (b). The precise T-DNA insertion location was obtained by sequencing of the amplicons from genomic DNA. (b) In both *bzip63-2* and -*3* mutants bZIP63 mRNA fragments were overrepresented downstream the T-DNA insertion compared to those detected upstream (two-tailed Student's *t*-test; n = 9; *$P < 0.05$). Values are means, and error bars indicate standard deviation. (c) Kinetics of *bZIP63* transcript decay in Ws and *bzip63-2* after treatment with the transcription inhibitor cordycepin. Plant growth, treatments and half-life calculation of *bZIP63* transcript were done as previously described (Matiolli *et al.*, 2011). The x-axis represents the elapsed time after treatment with cordycepin. The data were obtained from 3 biological replicates and each replicate consisted of the whole rosette collected from three plants (two-tailed Student's *t*-test; n = 9; *$P < 0.05$). Values are means, and error bars indicate standard deviation. Based on data from inhibited transcription, the increase of bZIP63 transcript levels in mutants *bzip63-2/-3*

is probably a consequence of an alteration in post-transcriptional regulation, resulting in greater bZIP63 transcript stability in these mutants. Gene expression data suggest that the bZIP63 chimeric transcript in these mutants is recognized as aberrant and is not translated or produces a non-functional protein, since the bZIP63 target genes *ASN1*, *DIN10* and *SEN1* (*Senescence-Associated Protein 1*) involved in the response to energy stress are downregulated in *bzip63-2/-3* just as in *bzip63-1* (Matiolli et al., 2011) and *bzip63* (Mair et a., 2015), a leaky and a knockout T-DNA insertion mutant, respectively. Several studies have reported increased levels of transcripts downstream and/or upstream of T-DNA insertion in Arabidopsis (Bertrand *et al.*, 2005; Xu *et al.*, 2007; Fojtová *et al.*, 2011), however, in cases where the T-DNA insertion is in the exon, the chimeric transcripts were not translated or the proteins were non-functional (Wang *et al.*, 2008). (d) Complementation of *bzip63-2* mutant by backcrossing with Ws, showing that a single functional *bZIP63* allele can restore *bzip63-2* rosette growth rates to those observed in Ws. The data were obtained from 30 biological replicates, differences were tested performing ANOVA and the different letters indicate statistically significant difference (Tukey's HSD test, $P < 0.05$). Values are means, and error bars indicate standard deviation. (e) Diurnal rhythmic accumulation of *bZIP63* transcript (data source: http://diurnal.mocklerlab.org). LDHH_SM and LDHH_ST represent Arabidopsis Columbia-0 plants with 29 and 35 days old, respectively, grown in soil under equinoctial conditions (12 h light / 12 h dark). Longday (16 h light / 8 h dark) and shortday (8 h light / 16 h dark) stands for 7 days old Arabidopsis Columbia-0 seedlings, grown in MS (Murashige-Skoog medium supplemented with 3% sucrose).

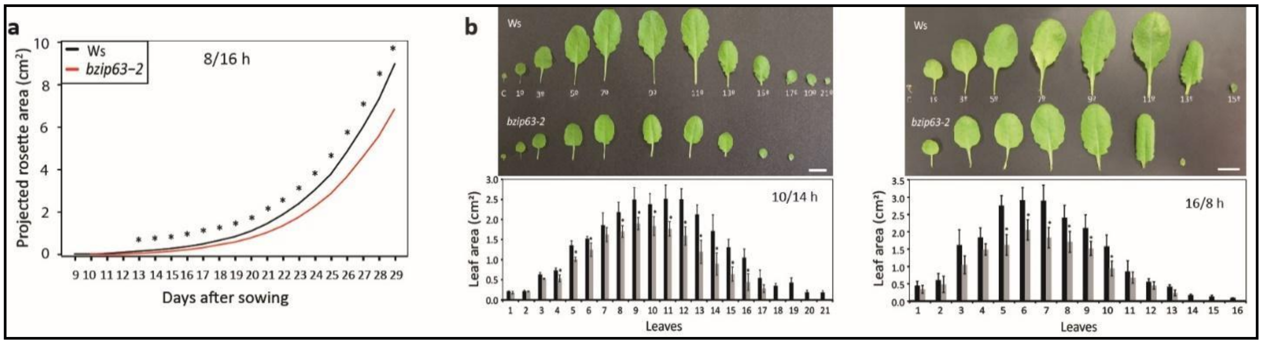

Fig. S2 *bzip63-2* mutant has a reduced leaf area and growth rate. (a) Daily records of *bzip63-2* and Ws rosette areas up to 29 days after sowing plants using the Phenoscope automated phenotyping system (https://phenoscope.versailles.inra.fr/; Tisné *et al.*, 2013), the impaired growth of *bzip63-2* was observed as early as 13 days after sowing. The data for each time point are means obtained from 14 and 8 biological replicates for Ws and *bzip63-2*, respectively. (b) The *bzip63-2* mutant has reduced leaf growth and development when grown in both short and long days photoperiod. In 30-day-old plants (stage of development 3.90; Boyes *et al.*, 2001) the leaf area of the *bzip63-2* mutant was nearly 30% and 20% smaller than Ws, whilst the leaves number were 17 and 13 for *bzip63-2* and 21 and 16 for Ws when grown in under short- and long-day photoperiod, respectively. The picture shows the odd numbered leaves. Asterisks denote a significant difference between the means using Student’s t-test (*$P < 0.05$). Values are means, and error bars indicate standard deviation. Bars, 1 cm.

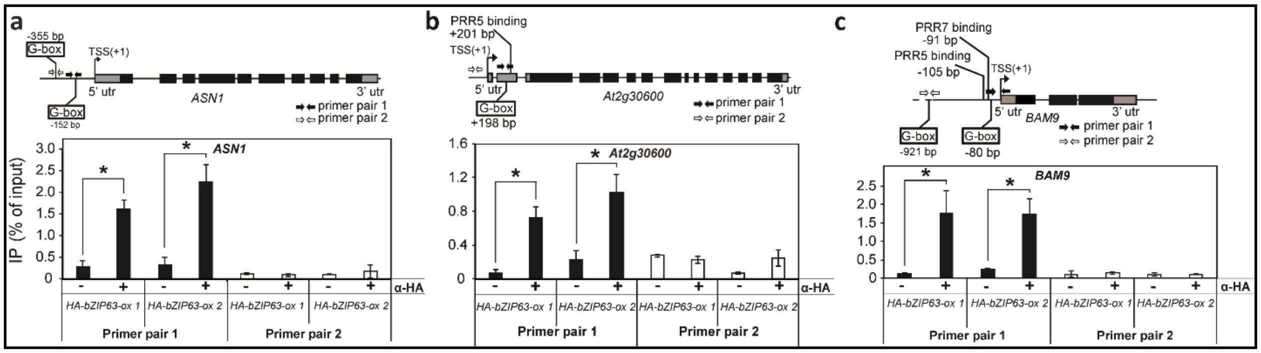

Fig. S3 bZIP63 binds to energy stress responsive genes. Enrichment of specific bZIP63-bound cis regulatory regions of genes related to energy stress responses in two independent transgenic lines: *HA-bZIP63*-ox1 in *bzip63-2* background, and *HA-bZIP63*-ox2 in Ws background. bZIP63 binding to (a) *ASN1*, (b) *AT2G30600* and (c) *BAM9* cis regulatory sequences were assessed by ChIP (Chromatin Immunoprecipitation) using anti-HA antibody followed by qPCR. The schemes of gene structure depict the binding sites of the circadian clock transcriptional regulators PRR7 and PRR5, as well as the bZIP63 binding region containing canonical G-box motifs. The numbers indicate the position of binding sites and motifs relative to the transcription start site (TSS+1) according to TAIR10 (www.arabidopsis.org). For ChIP-qPCR assays, shoot samples of 12-day old *HA-bZIP63*-ox1 and *HA-bZIP63*-ox2 plants were harvested at the end of the night (EN). Primer pair 1 amplifies the promoter region containing the closer G-box motif relative to the TSS+1, while primer pair 2 amplifies a region containing the second closer G-box motif of TSS+1 or without G-box or any putative bZIP binding site. - indicates mock and + indicates immunoprecipitated samples. Data were obtained from three independent experiments for *HA-bZIP63*-ox1 and six for *HA-bZIP63*-ox2 (Student's *t*-test; $*P < 0.05$). Values are means, and error bars indicate standard deviation.

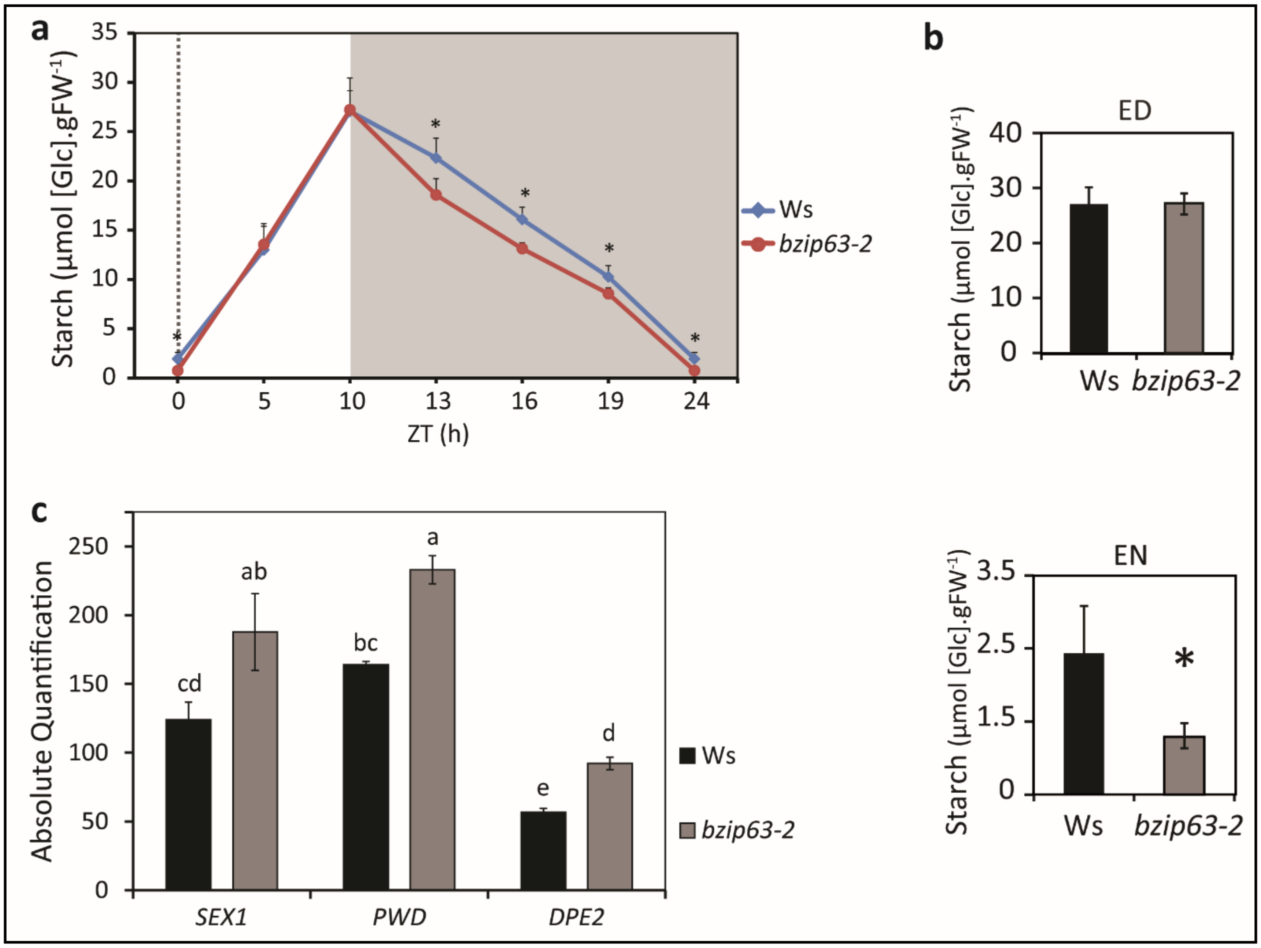

Fig. S4 *bzip63-2* starch degradation pattern under short days. (a) Time course analysis of starch levels in *bzip63-2* and Ws leaves through the diel cycle under SD conditions. Values are means, and error bars indicate standard deviation. (b) Starch levels at the end of the day (ED; ZT = 10 h) and EN (ZT = 24 h) in leaves of *bzip63-2* as compared to Ws. The average amount of starch for each time point was measured from five biological replicates, and each replicate consisted of leaves collected from six 30-day old plants (Student's *t*-test; n = 30; *$P < 0.05$). Values are means, and error bars indicate standard deviation. (c) The absolute transcript levels of starch degradation-related genes *SEX1, PWD* and *DPE2*, are higher in *bzip63-2* at EN under SD conditions. Data were obtained from one experiment with three biological replicates, and each replicate consisted of four youngest leaves and the shoot apical meristems collected from five 25-day old plants. Values are means, and error bars indicate standard deviation (n = 6), differences were tested performing ANOVA and the different letters indicate groups that are significantly different (Tukey's HSD test, $P < 0.05$).

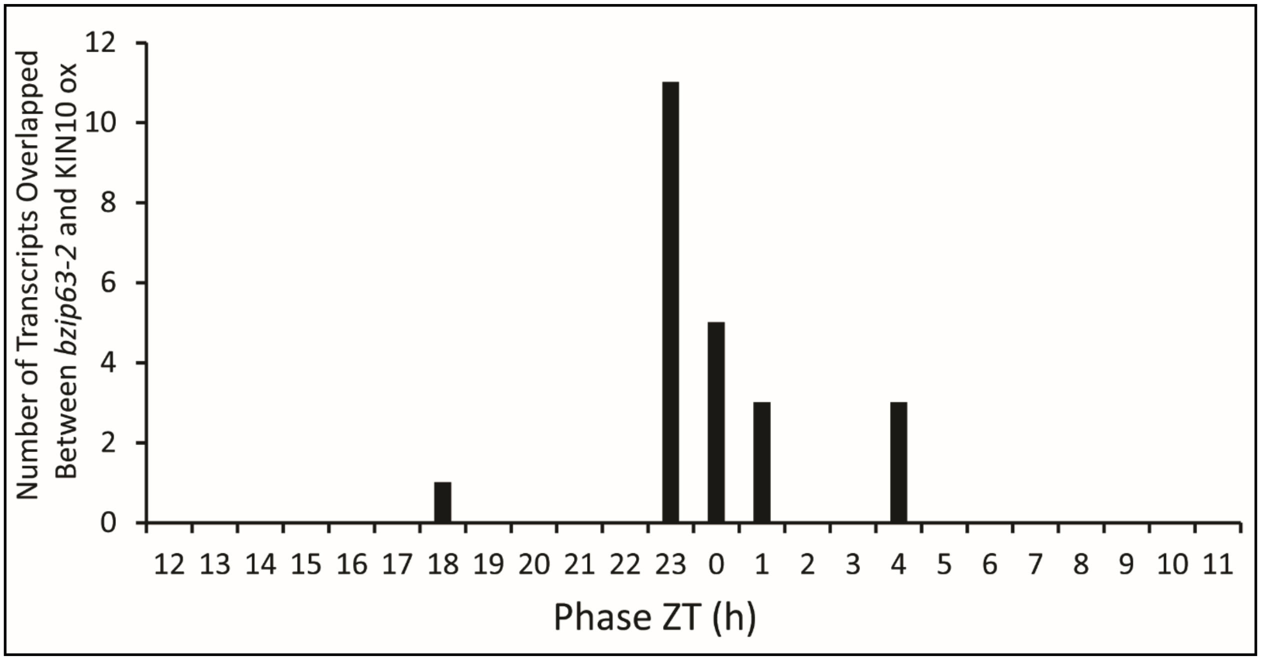

Fig. S5 Circadian phase of KIN10-induced energy-stress responsive genes. Circadian phase of the 27 downregulated genes in *bzip63*-2 mutant that overlap with genes induced by KIN10 overexpression and oscillate under entrainment conditions. An enrichment of genes oscillating with the same phase as *bZIP63* (ZT 23h) was observed. Circadian phase data was obtained from DIURNAL Database (http://diurnal.mocklerlab.org).

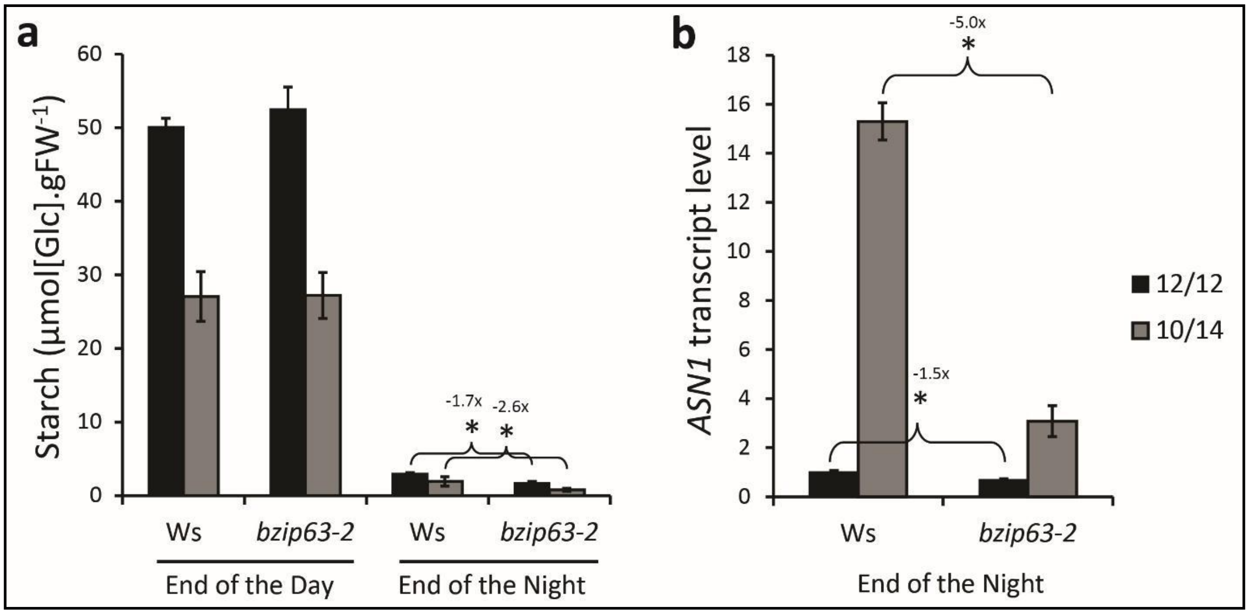

Fig. S6 Starch availability in short day versus 12-12 photoperiods. (a) Starch amounts in leaves of *bzip63-2* mutant compared to Ws grown under equinoctial or short day conditions, at ED (ZT = 12 h in 12-12; ZT = 10 h in short day) and EN (ZT = 24 h in 12-12 and short day). The average amount of starch was calculated from data obtained in two independent experiments, with five biological replicates, and each replicate consisted of leaves collected from six 30-day old plants cultivated in soil at 22º C and PAR = 100 µmol.m^{-2}.s^{-1} (Student's *t*-test; n = 60 for starch; *$P < 0.05$). Values are means, and error bars indicate standard deviation. (b) The transcript levels at EN of energy deficit marker gene *ASN1*, in *bzip63-2* mutant compared to Ws grown under 12-12 and short day photoperiods. Data were obtained from three independent experiments, with three biological replicates, and each replicate consisted of four youngest leaves and the shoot apical meristems collected from five 25-day old plants (Student's *t*-test; n = 9; *$P < 0.05$). Values are means, and error bars indicate standard deviation.

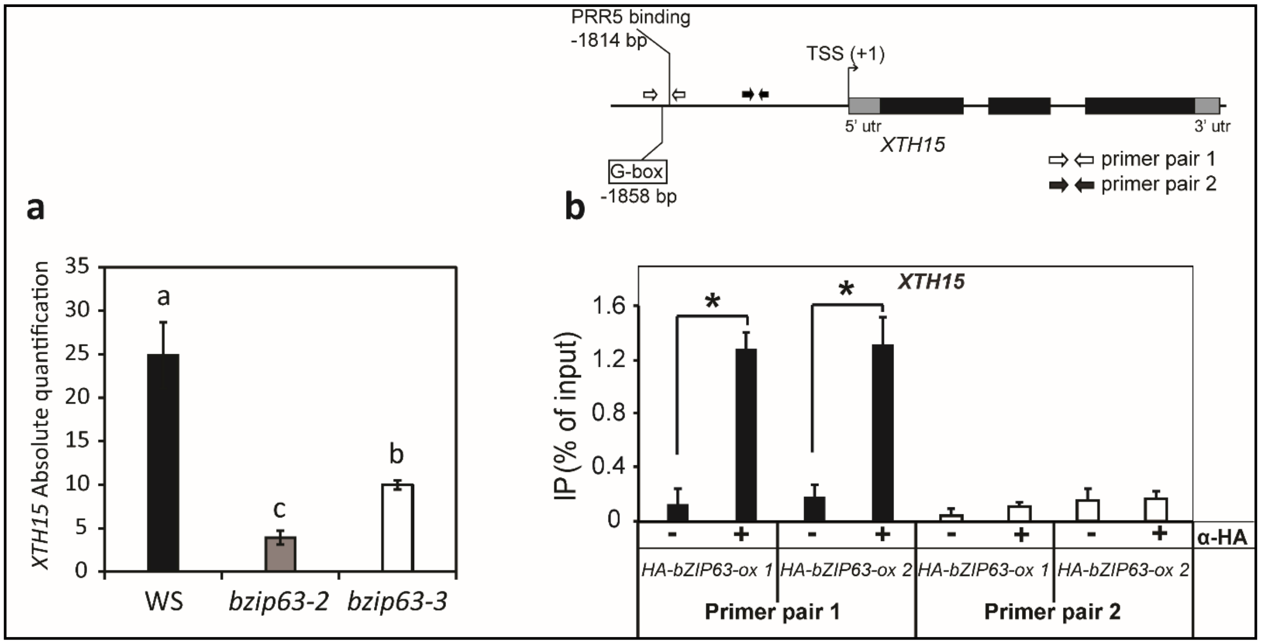

Fig. S7 bZIP63 binds to cell wall modification gene. (a) The absolute transcript levels of *XTH15*, which is involved in cell wall modification, are lower in *bzip63-2* and *bzip63-3* at EN. Data were obtained from three independent experiments, with three biological replicates, and each replicate consisted of four youngest leaves and the shoot apical meristems collected from five 25-day old plants, differences were tested performing ANOVA and the different letters indicate groups that are significantly different (Tukey's HSD test, $P < 0.05$). Values are means, and error bars indicate standard deviation. (b) Enrichment of specific bZIP63-bound cis regulatory regions of *XTH15* in two independent transgenic lines: *HA-bZIP63*-ox1 and *HA-bZIP63*-ox2. bZIP63 binding to *XTH15* cis regulatory sequences were assessed by ChIP as described in Supplementary Fig. 3. Data were obtained from three independent experiments for *HA-bZIP63*-ox1 and six for *HA-bZIP63*-ox2 (Student's *t*-test; $*P < 0.05$). Values are means, and error bars indicate standard deviation.

Table S1

Spreadsheet_Up bzip63-2 EN: Up-regulated genes in bzip63-2 plants compared with the wild type ($p < 0.05$) at the end of the night. Annotation was performed on TAIR10. Fold-change is ATH1 GeneChip.

Spreadsheet_Down bzip63-2 EN: Down-regulated genes in bzip63-2 plants compared with the wild type ($p < 0.05$) at the end of the night. Annotation was performed on TAIR10. Fold-change is ATH1.

Spreadsheet_Classes: Classes of differentially expressed genes in bzip63-2 mutant at the end of the night.

Table S2 Genes related to circadian clock, starch degradation and energy stress deregulated in bZIP63 mutants at end of the night (ZT24).

Biological Process	Gene name	AGI	*bzip63-2*[a]	*bzip63-3*[b]	*HA-bZIP63 ox2*[c]	*HA-bZIP63 ox1*[c]	HA-bZIP63 binding	CCA1 binding[d]	PRR7 binding[e]	PRR5 binding[e]	Oscilate in LL[f]
	**bZIP63**	AT5G28770	-	-	*2.6*	*4.2*	..	bound	bound	bound	Yes
Circadian clock	**PRR7**	AT5G02810	-1.5	-1.5	1.9	2.0	bound	bound	bound	bound	Yes
Energy stress-responsive genes	**ASN1**	AT3G47340	-4.2	-5.2	2.1	3.8	bound	not bound	not bound	not bound	Yes
	**BTB/POZ**	AT2G30600	-7.4	-3.0	1.7	6.0	bound	bound	not bound	bound	Yes
	**KMD4**	AT3G59940	-1.7	-1.3	1.2	1.6	..	bound	bound	bound	Yes
	**DIN10**	AT5G20250	-1.5	-1.6	1.1	1.0	..	bound	bound	bound	Yes
	**BAM9**	AT5G18670	-1.9	1.0	1.5	2.4	bound	not bound	bound	bound	Yes
	**BGAL4**	AT5G56870	5.3	3.6	1.2	1.1	not bound	not bound	not bound	not bound	No
	**DRM1**	AT1G28330	1.9	1.5	1.3	1.4	not bound	not bound	not bound	bound	Yes
Starch degradation-related genes	**SEX1**	AT1G10760	1.6	2.0	-1.4	-1.6	bound	bound	not bound	bound	Yes
	**PWD**	AT5G26570	1.5	1.4	1.0	1.0	not bound	bound	not bound	not bound	Yes
	**DPE2**	AT2G40840	1.5	1.9	2.0	2.0	not bound	not bound	not bound	not bound	Yes

(dark box) bound
(light box) not bound

.. Not tested in ChIP HA-bZIP63
a T-DNA insertion mutant (FLAG_610A08, Ws background) (Matiolli et al., 2011).
b T-DNA insertion mutant (FLAG_532A10, Ws background; Supplementary Fig. 1a-b).
c *HA-bZIP63*-ox2 were obtained in Ws, and *HA-bZIP63*-ox1 was obtained in *bzip63-2*.
d Data from Nagel et al., 2015, PNAS.
e Data from Liu et al., 2016, Plant Physiology.
f Data from Diurnal, Mockler et al., 2007.

Table S3 Relative metabolite content at EN in leaves of *bzip63-2* as compared to Ws.

Metabolite	Ws		*bzip63-2*		*P*
	avg nmol.gFW^{-1}	sdv	avg nmol.gFW^{-1}	sdv	
Lactate	1,23	0,20	1,22	0,12	0,959
2-Hydroxypyridine	1,11	0,17	1,19	0,22	0,593
Alanine	1,13	0,29	1,15	0,33	0,949
Pyruvate	0,93	0,20	0,87	0,10	0,723
Valine	0,94	0,22	0,88	0,22	0,720
Glycerol	1,18	0,15	1,39	0,33	0,312
Leucine	1,04	0,09	1,05	0,12	0,884
Isoleucine	0,92	0,14	1,05	0,13	0,201
Glycine	0,20	0,02	0,15	0,02	**0,017**
Orthophosphate	1,78	0,28	1,30	0,32	0,070
Proline	0,78	0,22	0,86	0,28	0,727
Glycerate	0,25	0,03	0,29	0,02	0,075
Benzoate	1,34	0,09	1,62	0,24	0,098
Serine	0,54	0,12	0,53	0,14	0,893
Succinate	1,96	0,39	1,55	0,29	0,104
Threonine	0,42	0,08	0,46	0,09	0,541
C4H4O4 [Fumarate|Maleate]	0,80	0,16	0,48	0,09	**0,003**
Nonanoate	1,07	0,22	1,40	0,31	0,221
Uracil	1,38	0,11	1,17	0,24	0,283
b-Alanine	0,89	0,12	1,03	0,15	0,187
Citramalate	1,06	0,15	1,03	0,08	0,789
Malate	0,35	0,07	0,25	0,05	**0,044**
trans-4-Hydroxyproline	0,96	0,17	1,06	0,08	0,356
4-Aminobutanoate	0,99	0,10	1,02	0,24	0,927
Aspartate	1,12	0,07	1,26	0,12	**0,044**
Asparagine (4TMS) MP	0,45	0,17	0,77	0,26	0,176
Methionine	0,67	0,11	0,62	0,11	0,577
Arginine	0,55	0,07	0,64	0,05	0,080
C5H10O5 [Xylose|Arabinose|Lyxose]	0,83	0,31	0,81	0,23	0,995
(r|x) Xylitol	0,86	0,19	0,93	0,10	0,590
(r|x) C5H10O5 [Ribose|Ribulose]	0,92	0,28	0,90	0,31	0,894
5-Oxoproline	0,92	0,07	1,00	0,07	0,126
Glutamate	1,17	0,13	1,05	0,12	0,197
(r|x) Putrescine	0,73	0,19	0,65	0,16	0,561
C6H12O5 [Fucose|Epifucose]	0,97	0,03	1,05	0,04	**0,032**
Ketoglutarate - 2OG	1,20	0,11	0,90	0,12	**0,035**
Glutamine	0,55	0,44	0,73	0,18	0,420
Phenylalanine	0,63	0,06	0,73	0,10	0,091
Asparagine	1,49	0,16	1,43	0,23	0,607
Agmatine	0,86	0,09	0,92	0,07	0,447
Ornithine	1,22	0,02	0,92	0,09	**0,002**
Fructose [|Psicose]	0,81	0,07	0,83	0,10	0,755

**Table S3. continuation**					
(r|x) Shikimate	0,88	0,12	0,92	0,12	0,618
Glucose	1,50	0,13	1,68	0,17	0,095
Citrate	1,55	0,16	1,72	0,16	0,126
Isocitrate	1,54	0,22	1,58	0,10	0,685
Citrulline	0,44	0,01	0,54	0,03	**0,003**
Tetradecanoate	1,08	0,60	1,33	0,58	0,578
Dehydroascorbate_MP	1,53	0,38	1,46	0,42	0,804
Gluconolactone	1,44	0,14	1,27	0,29	0,330
Dehydroascorbate	1,77	0,56	1,41	0,44	0,395
Tyramine	0,96	0,26	1,23	0,24	0,220
myo-Inositol	0,97	0,26	1,07	0,24	0,565
Tyrosine	0,97	0,06	0,97	0,05	0,999
C8H15NO6 [N-Acetyl-[Glc|Gal]]	1,10	0,36	0,88	0,11	0,458
Histidine	1,66	0,46	1,68	0,41	0,933
Adenine	0,95	0,06	1,18	0,07	**0,003**
(r|z) Spermidine	1,04	0,23	1,18	0,17	0,368
C6H13O9P [Glc 6-P|Gal 6-P]	1,20	0,11	0,92	0,05	**0,006**
Sucrose	0,56	0,05	0,48	0,09	0,135
(r|x) Maltose_best	3,06	0,21	1,75	0,15	**0,000**
(r|x) Maltose2	1,19	0,09	0,97	0,05	**0,002**
Trehalose	1,26	0,31	1,10	0,15	0,427
Galactinol	0,89	0,22	0,88	0,17	0,988
(r|x) Raffinose|1-Kestose|Inulotriose	0,62	0,15	0,62	0,23	0,983

Number in bold style are significantly different by Student’s *t*-test; $*P < 0.05$.

Table S4 List of primers used for quantification of the mRNA levels and immunoprecipitated sequences by RT-qPCR and amplification of genomic DNA.

**Primer name**	**Sequence 5′ - 3′**	**Function**	**Amplification efficiency E =[10^(–1/slope)-1]**
bZIP63a-Fw	TCTCCGACGAAGAAATCTCC	Quantify the mRNA levels by RT-qPCR. First exon	0.97
bZIP63a-Rv	GTATGAAACGATTGAATGCCC	Quantify the mRNA levels by RT-qPCR. First exon	
bZIP63b-Fw	GGAACTTTCATCAAACCTCAG	Quantify the mRNA levels by RT-qPCR. First and second exon-exon junction	0.94
bZIP63b-Rv	CACTGCTCATCATTGGTGTAG	Quantify the mRNA levels by RT-qPCR. Second exon	
bZIP63c-Fw	TGAACCCTACTAATGTTAAACG	Quantify the mRNA levels by RT-qPCR. Third exon	1.06
bZIP63c-Rv	CTGTGAAACTTGTGTCTCTAG	Quantify the mRNA levels by RT-qPCR. Fourth and fifth exon-exon junction	
bZIP63d-Fw	CTAGAGACACAAGTTTCACAG	Quantify the mRNA levels by RT-qPCR. Fourth and fifth exon-exon junction	1.03
bZIP63d-Rv	CTTCACTGTCTCTTCAGCCAT	Quantify the mRNA levels by RT-qPCR. Sixth exon	
bZIP63e-Fw	GCAAGGCCTTGATAGGGTG	Quantify the mRNA levels by RT-qPCR. Middle of sixth exon	0.98
bZIP63e-Rv	CTGATCCCCAACGCTTCGAA	Quantify the mRNA levels by RT-qPCR. End of sixth exon	
XTH15/XTR7 Fw	AACACATCATATTCTTGGTGGA	Quantify the mRNA levels by RT-qPCR	0.87
XTH15/XTR7 Rv	AGTAGATCCTCATGGGTTGAC	Quantify the mRNA levels by RT-qPCR	
XTH16 Fw	CCACAACACATCATATTTATGG	Quantify the mRNA levels by RT-qPCR	0.89
XTH16 Rv	GTAGCCCAATCATCTGCAT	Quantify the mRNA levels by RT-qPCR	
XTH33 Fw	CTCCCACCATACTGTATTTTTG	Quantify the mRNA levels by RT-qPCR	0.95
XTH33 Rv	GTTGACGGGGTACTTACCAC	Quantify the mRNA levels by RT-qPCR	
XTH27 Fw	CTCACATCATATTCTTTGTAGAC	Quantify the mRNA levels by RT-qPCR	1.04
XTH27 Rv	GTTGCCCATTTAGAACCGTC	Quantify the mRNA levels by RT-qPCR	
XTH6 Fw	CACATTGTTTTTTACGTAGACG	Quantify the mRNA levels by RT-qPCR	1.09
XTH6 Rv	CAGTCATCTGCTTCCCATAAT	Quantify the mRNA by RT-qPCR	
AT4G15620 Fw	ATGGAACACGAGGGCAAG	Quantify the mRNA by RT-qPCR	1.10
AT4G15620 Rv	TAAGGAAGCTTAATAGCGTC	Quantify the mRNA by RT-qPCR	
ATEXLA2 Fw	ATACAGGAGAGTTCCTTGCG	Quantify the mRNA by RT-qPCR	0.86
ATEXLA2 Rv	TGTAAATGGCTACCACTTCAG	Quantify the mRNA by RT-qPCR	
PRR7 Fw	TTCCGAAAGAAGGTACGATAC	Quantify the mRNA by RT-qPCR	1.06

Table S4. Continuation

PRR7 Rv	GCTATCCTCAATGTTTTTTATGT	Quantify the mRNA levels by RT-qPCR	
PGM Fw	ACCTTTAATCGATCTTGCATTG	Quantify the mRNA levels by RT-qPCR	1.05
PGM Rv	CATGTAATGACAGTGGGCTTC	Quantify the mRNA levels by RT-qPCR	
SEX1 Fw	CCTCTACGACAGTGTACCAA	Quantify the mRNA levels by RT-qPCR	1.07
SEX1 Rv	TCCAGCGCGTGCAATGTCT	Quantify the mRNA levels by RT-qPCR	
PYL5 Fw	GAGGCTCGAGATCTTGGACG	Quantify the mRNA levels by RT-qPCR	1.10
PYL5 Rv	CGCGTGTAGTGTCGTCACC	Quantify the mRNA levels by RT-qPCR	
PYL8 Fw	CTCCTGTTCATATTGTGTGGT	Quantify the mRNA levels by RT-qPCR	1.17
PYL8 Rv	ACTGTACCAATCTCCATGTTTC	Quantify the mRNA levels by RT-qPCR	
BAM9 Fw	GAGCACCAATCACCTGAATC	Quantify the mRNA levels by RT-qPCR	1.02
BAM9 Rv	CGACTCCTTGTTTCTTGCAG	Quantify the mRNA by RT- qPCR	
At4G15620 Fw	TGTCGGCATTTGTGTACAACA	Quantify the mRNA levels by RT-qPCR	1.10
At4G15620 Rv	TCTTGCTTCTTCTTCCCCTG	Quantify the mRNA levels by RT-qPCR	
At2g30600 Fw	AAAGCCTATGCGGGTACTTC	Quantify the mRNA levels by RT-qPCR	1.04
At2g30600 Rv	CTGATGTTCTTCGCCTAAGTC	Quantify the mRNA levels by RT-qPCR	
BGAL4 Fw	TTAACCTGGTACAAGTCTACG	Quantify the mRNA levels by RT-qPCR	0.93
BGAL4 Rv	GACGTCCAATATTTCTACCG	Quantify the mRNA levels by RT-qPCR	
RT_PP2AA3 Fw	CATGTTCCAAACTCTTACCTG	Quantify the mRNA levels by RT-qPCR	1.01
RT_PP2AA3 Rv	GTTCTCCACAACCGCTTGGT	Quantify the mRNA levels by RT-qPCR	
Actin2 Fw	CGTACAACCGGTATTGTGCTGG	Quantify the mRNA levels by RT-qPCR	0.98
Actin2 Rv	CTCTCTCTGTAAGGATCTTCATG	Quantify the mRNA levels by RT-qPCR	
PWD Fw	AGAAACAATCAACAGCATAAGC	Quantify the mRNA levels by RT-qPCR	1.10
PWD Rv	GATTGATTCATAGAGTCCTGC	Quantify the mRNA levels by RT-qPCR	
DPE2 Fw	ACTGGAGATACCGCGTACA	Quantify the mRNA by RT-qPCR	1.01
DPE2 Rv	CATTAGCAGGAACAGATCTTC	Quantify the mRNA by RT-qPCR	
AtSPS4F Fw	ACAAGCACTCAGGTATCTTTC	Quantify the mRNA by RT-qPCR	1.02
AtSPS4F Rv	GAATGATGGTTTTGTGGAGGC	Quantify the mRNA by RT-qPCR	
AtSPS1F Fw	AGCTCTGAGGTATTTGTTCG	Quantify the mRNA by RT-qPCR	0.98
AtSPS1F Rv	TGCACGACACTCCCTTTAG	Quantify the mRNA by RT-qPCR	
At3G59940 Fw	GGAAGGTAATTATGGATACGAT	Quantify the mRNA by RT-qPCR	0.95

Table S4. Continuation

At3G59940 Rv	CGTCGTCTTCACCATCATC	Quantify the mRNA levels by RT-qPCR	
At1G76410 Fw	ACATGGTTTCCACGTATCGT	Quantify the mRNA levels by RT-qPCR	0.97
At1G76410 Rv	GTAACCCGCCACATTTATGA	Quantify the mRNA levels by RT-qPCR	
PIF4 Fw	CACTGCAGTAAAACTGATAAAG	Quantify the mRNA levels by RT-qPCR	1.01
PIF4 Rv	ACTGCTGAGGTTGAACTCCG	Quantify the mRNA levels by RT-qPCR	
LHY Fw	AGAGGGTCGTATAGCGTTTC	Quantify the mRNA levels by RT-qPCR	1.16
LHY Rv	GCAGCACAAGAATCCTGGC	Quantify the mRNA levels by RT-qPCR	
CCA1 Fw	GTCTGACGAGGGTCGAATT	Quantify the mRNA levels by RT-qPCR	1.01
CCA1 Rv	CTGAGCTGTGAAGTTAAGATC	Quantify the mRNA levels by RT-qPCR	
GBS1 Fw	TGATCACTGCTGGAGCTGAC	Quantify the mRNA levels by RT-qPCR	0.95
GBS1 Rv	CCATATCTCATTGCGTGCAG	Quantify the mRNA levels by RT-qPCR	
ASN1 Fw	AGGTGCGGACGAGATCTTTG	Quantify the mRNA levels by RT-qPCR	0.95
ASN1 Rv	GTGAAGAGCCTTGATCTTGC	Quantify the mRNA levels by RT-qPCR	
KIN10 Fw	GATAACAACTACTTTGGAGAC	Quantify the mRNA levels by RT-qPCR	0.94
KIN10 Rv	CGAGTTTTATACAACTGAATTTC	Quantify the mRNA levels by RT-qPCR	
DRM1 Fw	GGAGAAGGGAGCAGCAGTA	Quantify the mRNA levels by RT-qPCR	1.05
DRM1 Rv	GAGTGGTTGGAGTCGTTGGA	Quantify the mRNA levels by RT-qPCR	
TOC1 Fw	CACATCTTTGCAGCATAGTCA	Quantify the mRNA levels by RT-qPCR	1.15
TOC1 Rv	TGTCAAGTTTATTTACCCTCAC	Quantify the mRNA levels by RT-qPCR	
CHE Fw	GGATTTCGGACAAGTCTGGA	Quantify the mRNA by RT-qPCR	1.04
CHE Rv	TGTTGCTGCTGGTGGCGGA	Quantify the mRNA by RT-qPCR	
DIN10 Fw	TGTCAGTGATTCTCCTGGAA	Quantify the mRNA by RT-qPCR	1.02
DIN10 Rv	CACCATCACGGGCAGGATC	Quantify the mRNA by RT-qPCR	
ProDH Fw	CGCTATACCGTATCTTCTCC	Quantify the mRNA by RT-qPCR	1.1
ProDH Rv	CTCTTAAGTTCCATCCTCATG	Quantify the mRNA by RT-qPCR	
SEX1 1 Fw	GGAGTCCACAGATACCGTCC	Quantify ChIP sequences by qPCR	1.04
SEX1 1 Rv	CGACACTTTATCCTTCGATTCCA	Quantify ChIP sequences by qPCR	
SEX1 2 Fw	ACGTGGTGGAATCGAAGGAT	Quantify ChIP sequences by qPCR	1.17
SEX1 2 Rv	GGGGTTTCTTCGCTCTTTGT	Quantify ChIP sequences by qPCR	
SEX1 3 Fw	CCATTCTCCTTTCTTGAACCCA	Quantify ChIP sequences by qPCR	0.99
SEX1 3 Rv	AGTTTGTGTTTGTCTTTGTCACT	Quantify ChIP sequences by qPCR	
ASN1 1 Fw	TGGAAGAAACCGGACAAAAG	Quantify ChIP sequences by qPCR	1.03

Table S4. Continuation

ASN1 1 Rv	ACCAGCTGTTTCCACGTGTT	Quantify ChIP sequences by qPCR	
ASN1 2 Fw	AATCAAATACACCATTGTCGTG	Quantify ChIP sequences by qPCR	1.1
ASN1 2 Rv	CGGTGTAAAAACTGTCCAATG	Quantify ChIP sequences by qPCR	
BAM9 1 Fw	TAACGACACGTGTAATGTCCAA	Quantify ChIP sequences by qPCR	0.91
BAM9 1 Rv	TTCGATTATGGACACAAAGGAG	Quantify ChIP sequences by qPCR	
BAM9 2 Fw	GGGAATATACTTGCATGGACCT	Quantify ChIP sequences by qPCR	0.97
BAM9 2 Rv	AATGAGAAAAAGCCACGTCA	Quantify ChIP sequences by qPCR	
XTH15 1 Fw	TGCCGGCTGGAATAGATAGA	Quantify ChIP sequences by qPCR	0.98
XTH15 1 Rv	ATGGCCACTCTTCTCCCTTT	Quantify ChIP sequences by qPCR	
XTH15 2 Fw	TCCATAAGAGAGAATGGTGTGG	Quantify ChIP sequences by qPCR	0.89
XTH15 2 Rv	TCGCATCCTCCTACATAGTGAT	Quantify ChIP sequences by qPCR	
AT2G30600 1 Fw	GCATCTGATTGTTTTGCTCGTG	Quantify ChIP sequences by qPCR	1.02
AT2G30600 1 Rv	CTGGCTTCATACCCCACCAT	Quantify ChIP sequences by qPCR	
AT2G30600 2 Fw	CGTATTCGTGTCTCTCATTTCCT	Quantify ChIP sequences by qPCR	0.89
AT2G30600 2 Rv	TCCGAGCGTACCTAATGAACT	Quantify ChIP sequences by qPCR	
BGAL 1 Fw	AGCTCAGAATATAGAGGCGCA	Quantify ChIP sequences by qPCR	0.93
BGAL 1 Rv	AAGTCAAGTTGCCGCATTCA	Quantify ChIP sequences by qPCR	
BGAL 2 Fw	TTCTTTAGGCAAGTCCGGT	Quantify ChIP sequences by qPCR	0.86
BGAL 2 Rv	TCATCTTGACTCTTGAGAACGAA	Quantify ChIP sequences by qPCR	
DRM1 1 Fw	TATGGTTGGATCTGACGGCC	Quantify ChIP sequences by qPCR	0.87
DRM1 1 Rv	TGGGAAGATGTCTCCATACGG	Quantify ChIP sequences by qPCR	
DRM1 2 Fw	TGGAAACCTTTTGTGCGACA	Quantify ChIP sequences by qPCR	1.14
DRM1 2 Rv	TCTTCTGAGTCAAAGCCGGT	Quantify ChIP sequences by qPCR	
DPE2 1 Fw	AGCCCTCTTCAATGGCGG	Quantify ChIP sequences by qPCR	1.13
DPE2 1 Rv	CGAGTAGTGCCAGCTGAAAG	Quantify ChIP sequences by qPCR	
DPE2 2 Fw	CGGTTTGTTCAAGCAAAGGT	Quantify ChIP sequences by qPCR	0.94
DPE2 2 Rv	AGAAATCAGCAACACGCAAG	Quantify ChIP sequences by qPCR	
DPE2 1b Fw	TCTTTGGTCTCACTTTGGTCTC	Quantify ChIP sequences by qPCR	0.95
DPE2 1b Rv	TGAGAGAGATAGAGAGTGAAGCA	Quantify ChIP sequences by qPCR	
DPE2 2b Fw	TGGACACTCTTGAGCGATCT	Quantify ChIP sequences by qPCR	0.95
DPE2 2b Rv	TTGTTCAATCGCTCTGCACC	Quantify ChIP sequences by qPCR	
bZIP63gen-Fw	ATCTCCGGTAACCATCACTG	Amplify *bZIP63* genomic DNA. First exon	-
bZIP63gen-Rv	CACCATTGTCAGATCTACCA	Amplify *bZIP63* genomic DNA. Second exon	-
Rb4	GGGTTGGGGTTTCTACAGAC	Annealing in the right border of T-DNA	-

Table S5 Circadian parameters calculated from two 24 h cycles under free-running conditions.

Gene Name	Genotype	Condition	Algorithm									
			COSOPT		BRASS		JTK Cycle			Meta Cycle 2D		
			**Period[1]**	pMMC-Beta	Period	Rel-Per	Adj.Pvalue	**Period[1]**	**LAG[1]**	Pvalue	**Phase[1]**	**Amplitude[1]**
CCA1	*bzip63-2*	LL	23.1	0.006	23.2	0.07	0.00004	24	0	0.0000	**23.1 ***	41.4
PRR7	*bzip63-2*	LL	22.3	0.022	23.3	0.09	0.02074	24	8	0.0004	7.1	**8.8 ***
TOC1	*bzip63-2*	LL	21.8	0.027	22.6	0.17	0.00155	24	14	0.0000	14.1	**3.2 ***
GBS1	*bzip63-2*	LL	23.1	0.010	22.8	0.09	0.00070	24	22	0.0000	21.7	23.6
SEX1	*bzip63-2*	LL	22.1	0.017	23.6	0.21	0.00320	28	**8 ***	0.0000	**8.3 ***	**2.5 ***
PWD	*bzip63-2*	LL	24.1	0.012	--	--	0.00030	24	14	0.0000	**12.3 ***	1.3
DPE2	*bzip63-2*	LL	22.2	0.059	--	--	0.14690	28	**6 ***	0.0273	**7.0 ***	1.0
bZIP63	*cca1/lhy*	LL	**16.6**	0.126	24.9	0.22	0.22258	**16**	4	0.0790	11.2	1.5
GBS1	*cca1/lhy*	LL	**15.2**	0.034	15.4	0.03	0.47480	**16**	8	0.6837	1.5	0.2
KMD4	*cca1/lhy*	LL	20.0	0.606	--	--	0.40185	28	4	0.0921	2.9	0.3
BAM9	*cca1/lhy*	LL	13.7	0.042	25.4	0.23	1	28	24	0.9421	21.3	0.4
CCA1	Ws	LL	24.1	0.008	24.2	0.11	0.00155	24	0	0.0000	23.8	43.8
PRR7	Ws	LL	22.4	0.020	22.6	0.14	0.00320	24	6.0	0.0000	7.5	15.2
TOC1	Ws	LL	22.3	0.015	22.4	0.15	0.00155	24	12.0	0.0000	12.6	6.9
GBS1	Ws	LL	24.1	0.024	24.2	0.17	0.00001	24	0.0	0.0000	23.4	26.8
SEX1	Ws	LL	22.6	0.021	22.7	0.22	0.02074	24	10.0	0.0006	10.8	3.9
PWD	Ws	LL	22.8	0.017	22.9	0.19	0.07661	24	14.0	0.0002	13.3	1.3
DPE2	Ws	LL	22.9	0.008	22.6	0.13	0.00237	24	8.0	0.0000	9.7	1.3
bZIP63	Ws	LL	**22.2**	0.038	24.1	0.16	0.00625	**24**	4.0	0.0001	4.9	2.6
GBS1	Ws	LL	**23.8**	0.047	24.2	0.21	0.00030	**24**	0.0	0.0000	23.5	5.3
KMD4	Ws	LL	22.5	0.026	22.4	0.18	0.02815	24	8.0	0.0005	9.4	1.0
BAM9	Ws	LL	23.1	0.017	25.0	0.24	0.00070	24	22.0	0.0000	22.8	1.3
CCA1	*bzip63-2*	LD	24.2	0.068	22.6	0.22	0.00003	24	0.0	0.0000	23.8	1181.4
PRR7	*bzip63-2*	LD	23.1	0.036	23.6	0.26	0.00057	24	8.0	0.0000	7.7	290.6
bZIP63	*cca1/lhy*	LD	24.6	0.027	22.7	0.11	0.03556	24	24.0	0.0006	23.0	**38.6 ***

Table S5 Continuation

GBS1	*cca1/lhy*	LD	23.8	0.021	23.0	0.23	0.00625	24	**22.0 ***	0.0002	**20.6 ***	5.2
KMD4	*cca1/lhy*	LD	24.8	0.090	--	--	0.00070	24	2.0	0.0001	0.4	1.9
BAM9	*cca1/lhy*	LD	25.6	0.029	24.5	0.22	0.03556	28	22.0	0.0003	21.6	**73.5 ***
CCA1	Ws	LD	25.4	0.058	25.5	0.21	0.00057	28	26.0	0.0000	24.7	991.2
PRR7	Ws	LD	23.0	0.014	23.5	0.23	0.00003	24	8.0	0.0000	7.7	333.5
bZIP63	Ws	LD	23.7	0.057	--	--	0.00001	24	2.0	0.0000	0.6	18.3
GBS1	Ws	LD	24.7	0.033	24.0	0.12	0.00112	24	0.0	0.0000	24.0	5.2
KMD4	Ws	LD	22.5	0.160	--	--	1	24	6.0	0.6725	6.0	0.6
BAM9	Ws	LD	24.5	0.048	13.8	0.06	0.00011	24	0.0	0.0000	23.8	44.0

[1] The circadian parameters period, phase (LAG) and amplitude were calculated using COSOPT and JTK Cycle to period, JTK Cycle and Meta Cycle to phase and amplitude. Numbers in bold style highlight bZIP63 and GBS circadian clock parameters in *cca1/lhy* double mutant and Ws or circadian clock and starch related genes in *bzip63-2* mutant.

* Significant thresholds with pMMC-Beta < 0.05 or P.value < 0.05.

Table S6 Circadian oscillator binding in downregulated genes in *bzip63-2* that are induced by KIN10 overexpression.

AGI	Name	CCA1 binding	PRR5 binding	PRR7 binding	PRR9 binding	TOC1 binding
AT1G02660	PLIP2	-	-	-	-	-
AT1G25400	AT1G25400	x	x	-	-	-
AT1G54740	DUF3049	-	x	-	-	-
AT1G62510	AT1G62510	-	-	-	-	-
AT1G68440	AT1G68440	-	x	x	x	x
AT1G69570	AT1G69570	x	x	x	x	-
AT1G80920	DNAJ8	x	x	-	-	-
AT2G17880	DNAJ PC24	-	x	-	-	-
AT2G30600	BTB/POZ	x	x	-	-	-
AT2G35170	AT2G35170	-	-	-	-	-
AT3G02140	TMAC2	-	x	x	-	-
AT3G07350	AT3G07350	-	x	x	-	-
AT3G26510	AT3G26510	-	x	-	-	-
AT3G29240	DUF179	-	x	-	-	-
AT3G47340	ASN1	-	-	-	-	-
AT3G47500	CDF3	-	x	x	-	-
AT3G57020	AT3G57020	-	x	x	-	-
AT3G59940	KMD4	x	x	x	-	-
AT3G61890	ATHB-12	-	x	x	x	x
AT4G03110	BRUNO-LIKE 1	-	-	-	-	-
AT4G35770	SEN1	-	-	-	-	-
AT4G37610	BT5_BTB	-	x	-	-	-
AT5G05440	PYL5	x	x	x	-	x
AT5G18670	BAM9	-	x	x	x	-
AT5G20250	DIN10	x	x	x	-	-
AT5G41080	AT5G41080	-	x	-	-	-
AT5G49360	BXL1	-	x	-	-	-

x Bound; **-** Not bound; [a]Data from Nagel et al., 2015, PNAS; [b]Data from Liu et al., 2016, Plant Physiology.

Supporting Information References

Bertrand C, Benhamed M, Li YF, Ayadi M, Lemonnier G, Renou JP, Delarue M, Zhou DX. **2005**. *Arabidopsis HAF2* gene encoding TATA-binding protein (TBP)-associated factor TAF1, is required to integrate light signals to regulate gene expression and growth. *Journal of Biological Chemistry* **280**: 1465–1473.

Boyes DC, Zayed AM, Ascenzi R, McCaskill AJ, Hoffman NE, Davis KR, Görlach J. **2001**. Growth stage-based phenotypic analysis of *Arabidopsis*: A model for high throughput functional genomics in plants. *Plant Cell* **13**: 1499–1510.

Fojtová M, Peška V, Dobšáková Z, Mozgová I, Fajkus J, Sýkorová E. **2011**. Molecular analysis of T-DNA insertion mutants identified putative regulatory elements in the *AtTERT* gene. *Journal of Experimental Botany* **62**: 5531–5545.

Liu TL, Newton L, Liu M-JJ, Shiu S-HH, Farre EM, Farré EM. **2016**. A G-box-like motif is necessary for transcriptional regulation by circadian pseudo-response regulators in *Arabidopsis*. *Plant Physiology* **170**: 528–539.

Matiolli CC, Tomaz JP, Duarte GT, Prado FM, del Bem LEV, Silveira AB, Gauer L, Corrêa LGG, Drumond RD, Viana AJ, *et al.* 2011. The Arabidopsis bZIP gene AtbZIP63 is a sensitive integrator of transient abscisic acid and glucose signals. *Plant Physiology* **157**.

Mockler TC, Michael TP, Priest HD. **2007**. The Diurnal project : diurnal and circadian expression profiling , model-based pattern matching , and promoter analysis. *CSH Symposia* **LXXII**: 353–363.

Nagel DH, Doherty CJ, Pruneda-Paz JL, Schmitz RJ, Ecker JR, Kay SA. **2015**. Genome-wide identification of CCA1 targets uncovers an expanded clock network in *Arabidopsis*. *Proceedings of the National Academy of Sciences of the United States of America* **112**: E4802–E4810.

Tisné S, Serrand Y, Bach L, Gilbault E, Ben Ameur R, Balasse H, Voisin R, Bouchez D, Durand-Tardif M, Guerche P, *et al.* 2013. Phenoscope: An automated large-scale phenotyping platform offering high spatial homogeneity. *Plant Journal* **74**: 534–544.

Wang YY, Yang Y, Chen Q, Yu J, Hou Y, Han L, He J, Jiao D, Yu H. **2008**. Simultaneous knockdown of p18INK4C, p27Kip1 and MAD1 via RNA interference results in the expansion of long-term culture-initiating cells of murine bone marrow cells in vitro. *Acta Biochimica et Biophysica Sinica* **40**: 711–720.

Xu XM, Rose A, Muthuswamy S, Jeong SY, Venkatakrishnan S, Zhao Q, Meier I. **2007**. NUCLEAR PORE ANCHOR, the Arabidopsis Homolog of Tpr/Mlp1/Mlp2/Megator, is involved in mrna export and sumo homeostasis and affects diverse aspects of plant development. *the Plant Cell Online* **19**: 1537–1548.
